# T-cell CX3CR1 expression as a dynamic blood-based biomarker of response to immune checkpoint inhibitors

**DOI:** 10.1038/s41467-021-21619-0

**Published:** 2021-03-03

**Authors:** Takayoshi Yamauchi, Toshifumi Hoki, Takaaki Oba, Vaibhav Jain, Hongbin Chen, Kristopher Attwood, Sebastiano Battaglia, Saby George, Gurkamal Chatta, Igor Puzanov, Carl Morrison, Kunle Odunsi, Brahm H. Segal, Grace K. Dy, Marc S. Ernstoff, Fumito Ito

**Affiliations:** 1 Center for Immunotherapy, Roswell Park Comprehensive Cancer Center, Buffalo, NY USA; 2 Department of Medicine, Roswell Park Comprehensive Cancer Center, Buffalo, NY USA; 3 Department of Medicine, University at Buffalo Jacobs School of Medicine and Biomedical Sciences, The State University of New York, Buffalo, NY USA; 4 Department of Biostatistics, Roswell Park Comprehensive Cancer Center, Buffalo, NY USA; 5 Department of Cancer Genetics and Genomics, Roswell Park Comprehensive Cancer Center, Buffalo, NY USA; 6 Department of Pathology, Roswell Park Comprehensive Cancer Center, Buffalo, NY USA; 7 Department of Gynecologic Oncology, Roswell Park Comprehensive Cancer Center, Buffalo, NY USA; 8 Department of Immunology, Roswell Park Comprehensive Cancer Center, Buffalo, NY USA; 9 Department of Surgical Oncology, Roswell Park Comprehensive Cancer Center, Buffalo, NY USA; 10 Department of Surgery, University at Buffalo Jacobs School of Medicine and Biomedical Sciences, The State University of New York, Buffalo, NY USA; 11 Present Address: Merck Sharp & Dohme, Tokyo, Japan; 12 Present Address: University of Chicago Comprehensive Cancer Center, Chicago, IL USA; 13 Present Address: Division of Cancer Treatment and Diagnosis, Developmental Therapeutics Program, National Cancer Institute, Bethesda, MD USA

**Keywords:** Immunotherapy, Tumour immunology

## Abstract

Immune checkpoint inhibitors (ICI) have revolutionized treatment for various cancers; however, durable response is limited to only a subset of patients. Discovery of blood-based biomarkers that reflect dynamic change of the tumor microenvironment, and predict response to ICI, will markedly improve current treatment regimens. Here, we investigate CX3C chemokine receptor 1 (CX3CR1), a marker of T-cell differentiation, as a predictive correlate of response to ICI therapy. Successful treatment of tumor-bearing mice with ICI increases the frequency and T-cell receptor clonality of the peripheral CX3CR1^+^CD8^+^ T-cell subset that includes an enriched repertoire of tumor-specific and tumor-infiltrating CD8^+^ T cells. Furthermore, an increase in the frequency of the CX3CR1^+^ subset in circulating CD8^+^ T cells early after initiation of anti-PD-1 therapy correlates with response and survival in patients with non-small cell lung cancer. Collectively, these data support T-cell CX3CR1 expression as a blood-based dynamic early on-treatment predictor of response to ICI therapy.

## Abstract (web summary)

There is an urgent need to discover blood-based biomarkers to predict response to immune checkpoint inhibitors (ICI). Here the authors show that effective ICI therapy correlates with increased frequency of circulating CX3CR1^+^CD8^+^ T cells in preclinical tumor models and in a cohort of patients with non-small cell lung cancer treated with anti-PD-1.

## Introduction

Cancer immunotherapies that target the immune checkpoints, such as cytotoxic T lymphocyte-associated antigen 4 (CTLA-4), programmed cell death protein-1 (PD-1), and PD1 ligand-1 (PD-L1), have transformed the therapeutic landscape of a variety of malignancies^1–3^. However, despite compelling clinical responses seen across diverse tumor types, only a fraction of patients achieve durable responses. Moreover, unusual response patterns such as pseudoprogression and delayed response, a dichotomous outcome, potentially severe toxicity, and high cost indicate a critical need for a reliable predictive biomarker^4–7^.

Baseline PD-L1 expression on immune and tumor cells, preexisting infiltrating CD8^+^ T cells, and tumor mutational burden (TMB) correlate with response^2–11^; however, the use of these pretreatment markers are hampered by the significant overlap between responders and non-responders, limited quantity and quality of the tissue, and/or lack of standardization^12–14^. Analysis of serially collected tumor samples could aid in the assessment of the evolution of the tumor microenvironment (TME) during immune checkpoint inhibitor (ICI) therapy^15–18^; however, this approach is invasive and challenging for visceral tumors such as non-small cell lung cancer (NSCLC). The discovery of dynamic circulating immune biomarkers that reflect the evolution of adaptive immunity in the TME and are early predictors of clinical response to ICI would be of value to guide the selection of patients most likely to benefit from ICI therapy.

Emerging blood-based biomarkers such as exosomal PD-L1, TMB, and T-cell receptor (TCR) sequence in cell-free DNA, and hypermutated circulating tumor DNA associate with response^19–23^; however, these approaches require complex platforms, and/or bioinformatics analysis, that limit their widespread application in community-based clinical practice. Since ICI targets T-cell regulatory pathways, the utility of surface and intracellular proteins expressed on T cells have been investigated as a potential biomarker for response^8,9,24–28^. Of these, the proliferation marker Ki-67 has been extensively investigated. However, most studies showed Ki-67 expression only transiently increased in subsets of peripheral blood (PB) CD8^+^ T cells after the first cycle with unclear predictive and prognostic value as a stand-alone biomarker for ICI^8,9,24–27^.

Recently, CX3C chemokine receptor 1 (CX3CR1) was found to be a marker of T-cell differentiation, where CX3CR1^+^ CD8^+^ T cells were the progeny of CX3CR1^−^CD8^+^ T cells, and exhibited robust cytotoxicity in anti-viral immunity^29,30^. Mechanistically, CX3CR1 is stably expressed on CD8^+^ T cells through unidirectional differentiation from CX3CR1^−^CD8^+^ T cells during the effector phase^30,31^, which theoretically provides an advantage as a biomarker compared with transiently expressed molecules on T cells. Indeed, increased frequency of PB CX3CR1^+^ CD8^+^ T cells has been observed in a few patients who responded to anti-VEGF and anti-PD-L1 antibodies (Ab) for renal cell carcinoma^32^. Furthermore, Yan et al.^33^ have reported the increased frequency of CX3CR1^+^ granzyme B^+^ T cells among PB CD8^+^ T cells in melanoma patients who responded to anti-PD-1 Ab compared to non-responders. Collectively, evidence from preclinical and clinical studies prompted us to evaluate the role of CX3CR1 as a blood-based T-cell biomarker of response to ICI therapy.

Here, we hypothesize that changes in the frequency of PB CX3CR1^+^ CD8^+^ T cells would correlate with response to ICI and help identify responders vs. non-responders early after initiation of therapy. We investigate the frequency of CX3CR1^+^ CD8^+^ T cells in PB before and during ICI therapy, and delineate the TCR repertoire in peripheral CX3CR1^+^ CD8^+^ T-cell subsets and CD8^+^ tumor-infiltrating lymphocytes (TILs) using preclinical models. To understand the clinical utility of CX3CR1 as a circulating T-cell biomarker, we analyze longitudinal PB samples from patients with NSCLC undergoing anti-PD-1 therapy and evaluate changes in the frequency of PB CX3CR1^+^ CD8^+^ T cells as a correlate of response to anti-PD-1 therapy. Our results support circulating CX3CR1^+^ CD8^+^ T cells as an early on-treatment biomarker of clinical response to anti-PD-1 therapy.

## Results

### Effective ICI therapy correlates with increased frequency of circulating CX3CR1^+^ CD8^+^ T cells

**Fig. 1:**
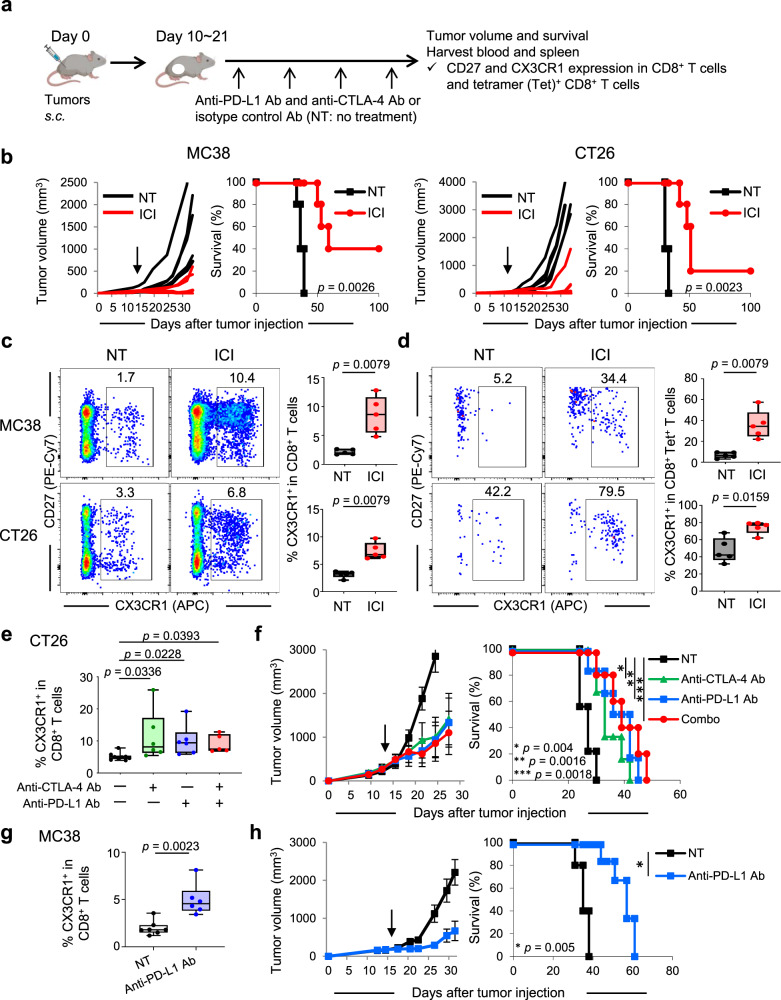
Effective immune checkpoint inhibitor therapy correlates with the increased frequency of circulating CX3CR1^+^ CD8^+^ T cells. **a** Experimental scheme of treatment with immune checkpoint inhibitors (ICI). **b** Individual tumor growth and survival curves in MC38 and CT26 tumor-bearing mice treated with isotype antibody (Ab) (NT) or anti-PD-L1 Ab and anti-CTLA-4 Ab (ICI). **c**, **d** Representative flow cytometry plots and data panel showing the frequency of CX3CR1^+^ cells among CD8^+^ T cells (**c**) and tetramer (Tet)^+^ CD8^+^ T cells (**d**) in peripheral blood (PB) of MC38 and CT26 tumor-bearing mice in different treatments as indicated. Numbers denote percent CX3CR1^+^ cells. The gating strategy is presented in Supplementary Fig. 1. PB was harvested 2 weeks after initiation of the treatment. *n* = 5 mice in all groups (**b**–**d**). **e**, **f** Frequency of the PB CX3CR1^+^ subset among CD8^+^ T cells (**e**), tumor growth curves (mean) and survival curves (**f**) in CT26-bearing mice in different treatment groups as indicated. *n* = 9 mice (NT), 6 mice (anti-CTLA-4 Ab), 6 mice (anti-PD-L1 Ab), and 5 mice (combo). **g** Frequency of the PB CX3CR1^+^ subset among CD8^+^ T cells in MC38 tumor-bearing mice treated with isotype control Ab (NT) or anti-PD-L1 Ab. *n* = 7 mice (NT) and 6 mice (anti-PD-L1 Ab). **h** Tumor growth curves (mean) and survival curves in MC38-bearing mice in different treatment groups as indicated. *n* = 5 mice in all groups. Data shown in **b**–**h** are representative of two independent experiments. PB was harvested 1 week after initiation of the treatment (**e**, **g**). Arrows indicate initiation of treatment (**b**, **f**, **h**). *P-*values were determined by a log-rank (Mantel–Cox) test (**b**, **f**, **h**), a two-tailed Mann–Whitney *U*-test (**c**, **d**, **g**) or Kruskal–Wallis with Dunn’s multiple comparisons (**e**). Data in **f**, **h** are presented as mean ± SEM. Box plots: dot, single PB; hinges, 25th and 75th percentiles; middle line, median; whiskers, minimum to maximum value (**c**–**e**, **g**). Source data are provided as a Source Data file.

While ICI is known to restore cytokine production and proliferation of effector T cells^34^, a recent study in a murine infection model indicates that ICI also facilitates effector differentiation of T cells^35^. To further elucidate this mechanism, we evaluated CX3CR1 expression, a marker of T-cell differentiation in combination with CD27 ^29–31^, on PB CD8^+^ T cells before and during treatment with anti-PD-L1 and anti-CTLA-4 Ab or isotype Ab (NT: no-treatment) in two mouse tumor models, MC38 and CT26 colon adenocarcinoma (Fig. 1a and Supplementary Fig. 1). Markedly improved tumor control and survival (Fig. 1b) with increased frequency of CX3CR1^+^ CD8^+^ T cells (Fig. 1c) were observed in MC38 and CT26 tumor-bearing mice treated with ICI compared to mice receiving isotype Ab. Next, we used a tetramer (Tet) to detect CD8^+^ T cells specific for mutated Adpgk protein (Adpgk^Mut^) in MC38 and shared tumor-associated antigen (TAA), gp70 in CT26 tumors^36,37^, and found substantially increased frequency of CX3CR1^+^ Tet^+^ CD8^+^ T cells in both tumor models (Fig. 1d), suggesting that T-cell differentiation after ICI therapy occurs in tumor-specific CD8^+^ T cells. We also tested whether the increase of PB CX3CR1^+^ CD8^+^ T cells can be seen in mice bearing B16 tumors which exhibit primary resistance to CTLA-4 or PD-1/PD-L1 blockade therapy^38,39^. There was a non-significant trend toward increased PB CX3CR1^+^ CD8^+^ T cells in B16 tumor-bearing mice without improvement of survival by combined CTLA-4/PD-L1 blockade therapy (Supplementary Fig. 2).

Although both anti-PD-L1 Ab and anti-CTLA-4 Ab target subsets of exhausted-like CD8^+^ T cells, they do so through distinct cellular mechanisms^40^. Therefore, we evaluated CX3CR1 expression of PB CD8^+^ T cells in CT26-bearing mice treated with either anti-PD-L1 Ab, anti-CTLA-4 Ab, or both. Increased frequency of the PB CX3CR1^+^ CD8^+^ T cells was seen after either monotherapy or combined ICI therapy compared to no treatment (Fig. 1e). Of note, there was no obvious difference in the antitumor efficacy between monotherapy and combined therapy (Fig. 1f), consistent with the frequency of PB CX3CR1^+^ CD8^+^ T cells. The increase of circulating CX3CR1^+^ CD8^+^ T cells was also observed in MC38 tumor-bearing mice treated with anti-PD-L1 therapy alone with improved tumor control and survival (Fig. 1g, h). Collectively, these findings suggest that effective ICI therapy correlates with increased frequency of PB CX3CR1^+^ CD8^+^ and CX3CR1^+^ Tet^+^ CD8^+^ T cells in mice.

### Peripheral CX3CR1^+^ CD8^+^ T cells display properties of recently activated effector cells with decreased capacity of trafficking across tumor vessels

**Fig. 2:**
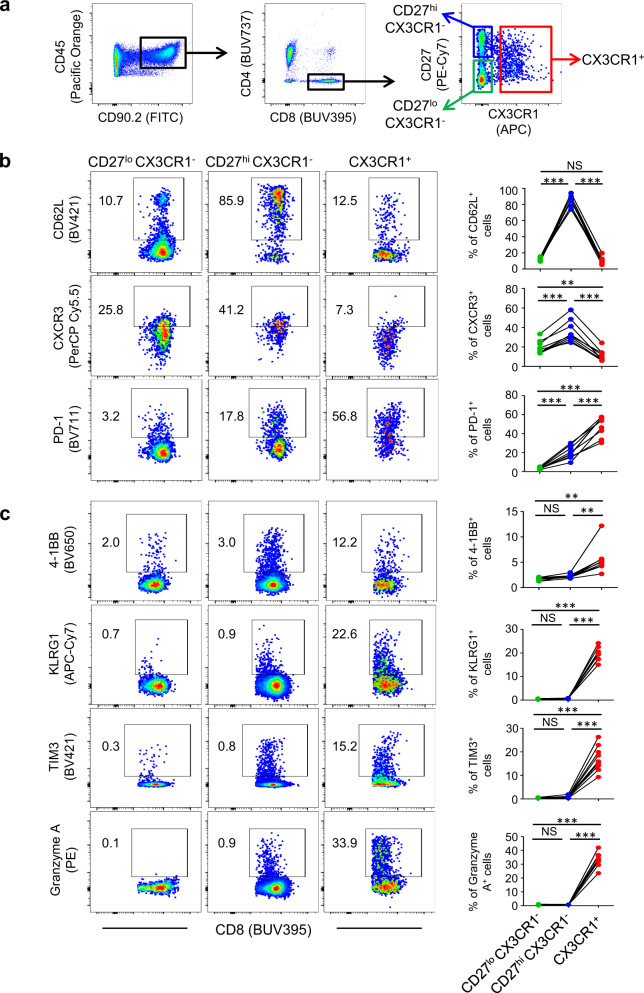
Phenotypic analysis of peripheral CD8^+^ T cells in mice treated with immune checkpoint inhibitor (ICI) therapy. **a** Gating strategy for phenotypic analysis of three subsets (CD27^lo^ CX3CR1^−^, CD27^hi^ CX3CR1^−^, and CX3CR1^+^) of peripheral CD8^+^ T cells in mice. **b**, **c** Mice bearing 10-day established CT26 tumors were treated with anti-PD-L1 antibody (Ab) and anti-CTLA-4 Ab every 3 days and every other day, respectively. Peripheral blood (PB) (**b**) and spleen (**c**) were harvested 2 weeks after initiation of the treatment. Representative flow-cytometric plots of three subsets (CD27^lo^ CX3CR1^−^, CD27^hi^ CX3CR1^−^, and CX3CR1^+^) of PB (**b**) and splenic (**c**) CD8^+^ T cells are shown. Data panels show frequency among CD8^+^ T cells. NS, not significant, *P < 0.05, **P < 0.005, ***P < 0.0001, by one-way repeated measures ANOVA with Tukey’s multiple comparisons (**b**, **c**). *n* = 9 mice in all groups (**b**, **c**). Data shown **b**, **c** are representative of two independent experiments. Source data are provided as a Source Data file.

Next, using a gating strategy based on the expression of CD27 and CX3CR1, we characterized the three subsets of CD8^+^ T cells (CD27^lo^ CX3CR1^−^, CD27^hi^ CX3CR1^−^ and CX3CR1^+^) in mice treated with ICI therapy (Fig. 2a). In CT26 tumor-bearing mice treated with CTLA-4 and PD-L1 blockade therapy, PB CX3CR1^+^ CD8^+^ T cells had low expression of L-selectin (CD62L) and CXCR3, trafficking receptors required for entry of blood-borne T cells across lymphoid organ high endothelial venules (HEV) and the tumor microvasculature, respectively (Fig. 2b)^41,42^. These findings are in parallel to our recent study and others showing that peripheral virus- and tumor-specific CD8^+^ T cells expressing high levels of CX3CR1 exhibit decreased CD62L and CXCR3 expression, and are predominantly located within the circulation^30,43^. Of note, the CX3CR1^+^ fraction was largely positive for PD-1 which is reported to be expressed in PB neoantigen-specific CD8^+^ T cells in patients^44^. Further profiling of three subsets of CD8^+^ T cells in CT26 tumor-bearing in Balb/c mice treated with anti-CTLA-4/PD-L1 therapy and MC38 tumor-bearing C57BL/6 mice treated with anti-PD-L1 monotherapy in spleen revealed that the CX3CR1^+^ subset was notable for increased expression of granzyme A, 4-1BB, TIM3, and KLRG1 regardless of mouse strain, tumor types, and monotherapy or combination ICI therapy (Fig. 2c and Supplementary Fig. 3), and that CX3CR1^+^ CD8^+^ T cells represent a subset of recently activated effector cells in agreement with prior studies^29–31,33^.

### CX3CR1 but not Ki-67 is stably upregulated in PB CD8^+^ T cells during ICI therapy

**Fig. 3:**
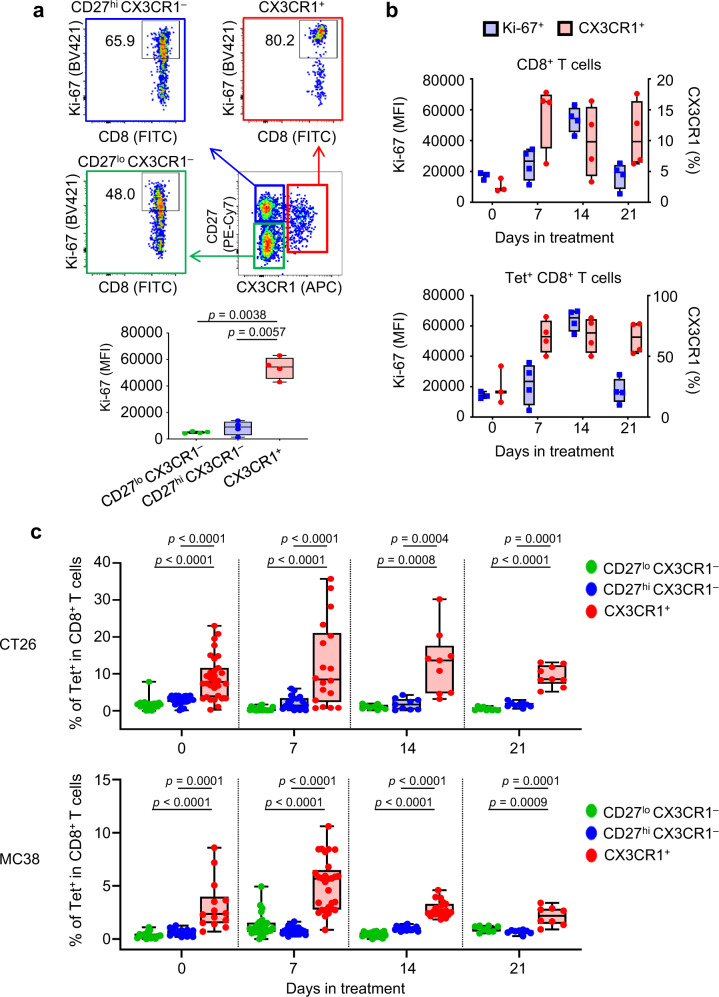
Phenotypic analysis of PB CX3CR1^+^ CD8^+^ T cells before and during ICI therapy. **a**–**c** CT26 (**a**–**c**) or MC38 (**c**) tumor-bearing mice were treated with anti-PD-L1 Ab and anti-CTLA-4 antibody (Ab) every 3 days and every other day, respectively. Peripheral blood (PB) was obtained before and 1, 2, and 3 weeks after the initiation of the treatment. Gating strategy: all cells > size lymphocytes > singlets > live > CD3^+^ CD8^+^ > CD27, CX3CR1. **a** Ki-67 expression of CD27^lo^ CX3CR1^−^ (green), CD27^hi^ CX3CR1^−^ (blue), and CX3CR1^+^ (red) CD8^+^ T cells in PB 2 weeks after ICI therapy. Numbers denote percent Ki-67^+^ cells. The data panel shows the median fluorescence intensity (MFI) of Ki-67^+^ cells in each subset. *n* = 4 mice in all groups. **b** Box and whiskers plots showing MFI of Ki-67^+^ (blue) and frequency (red) of the CX3CR1^+^ subset in CD8^+^ T cells (upper) and Tet^+^ CD8^+^ T cells (lower) at days 0, 7, 14, and 21 in PB. *n* = 3 mice (day 0) and *n* = 4 mice (days 7, 14, and 21). **a**, **b** Data shown are representative of two independent experiments. **c** Frequency of PB Tet^+^ CD8^+^ T cells in the CD27^lo^ CX3CR1^−^ (green), CD27^hi^ CX3CR1^−^ (blue), and CX3CR1^+^ (red) subsets before and 1, 2, and 3 weeks after the initiation of the treatment in CT26 (upper) and MC38 (lower) tumor-bearing mice. For CT26 tumor-bearing mice, *n* = 31 (day 0), *n* = 18 (day 7), *n* = 9 (day 14), and *n* = 9 (day 21) derived from three independent experiments. For MC38 tumor-bearing mice, *n* = 14 (day 0), *n* = 29 (day 7), *n* = 19 (day 14), and *n* = 8 (day 21) derived from three independent experiments. *P*-values were determined by one-way repeated measures ANOVA with Tukey’s multiple comparisons (**a**, **c**). Box plots: dot, single PB; hinges, 25th and 75th percentiles; middle line, median; whiskers, minimum to maximum value (**a**–**c**). Source data are provided as a Source Data file.

To gain further insight into the PB CX3CR1^+^ CD8^+^ T cells, we evaluated the expression of the nuclear protein Ki-67, a marker for proliferation that is upregulated in subsets of PB CD8^+^ T cells in response to ICI^8,9,24–27^. The levels of Ki-67 expression in the CX3CR1^+^ CD8^+^ T cells were significantly higher than in the CX3CR1^−^(CD27^lo^ CX3CR1^−^ and CD27^hi^ CX3CR1^−^) subsets 2 weeks after anti-CTLA-4/PD-L1 therapy in CT26 tumor-bearing mice (Fig. 3a). We next examined whether increased expression of CX3CR1 on PB CD8^+^ T cells is transient or sustained during anti-CTLA-4/PD-L1 therapy. Increased frequency of CX3CR1^+^ subset was seen in both CD8^+^ and Tet^+^ CD8^+^ T cells starting from day 7, which remained high during treatment (Fig. 3b). In contrast, Ki-67 expression peaked at day 14, and returned to the baseline at day 21 (Fig. 3b).

### Tumor-specific CD8^+^ T cells are enriched in the CX3CR1^+^ subset in PB

Identification of circulating T-cell biomarkers that enrich tumor-reactive T cells may facilitate the discovery of dynamic predictive markers of response to ICI. First, we assessed the frequency of Tet^+^ CD8^+^ T cells within the PB CX3CR1^+^ and CX3CR1^−^ subsets in MC38 and CT26 tumor-bearing mice (Supplementary Fig. 4). We found more Tet^+^ CD8^+^ T cells in the CX3CR1^+^ subset than in the CX3CR1^−^ subsets even before treatment although the frequency varied between individual mice (Fig. 3c). Moreover, the frequency of Tet^+^ CD8^+^ T cells remained higher in the CX3CR1^+^ subset than in the CX3CR1^−^ subsets in both tumor models (Fig. 3c), suggesting that the PB CX3CR1^+^ subset is enriched with tumor-specific CD8^+^ T cells.

### Clonally expanded TCR repertoires of CD8^+^ TILs are enriched in the peripheral CX3CR1^+^ subset during ICI therapy

**Fig. 4:**
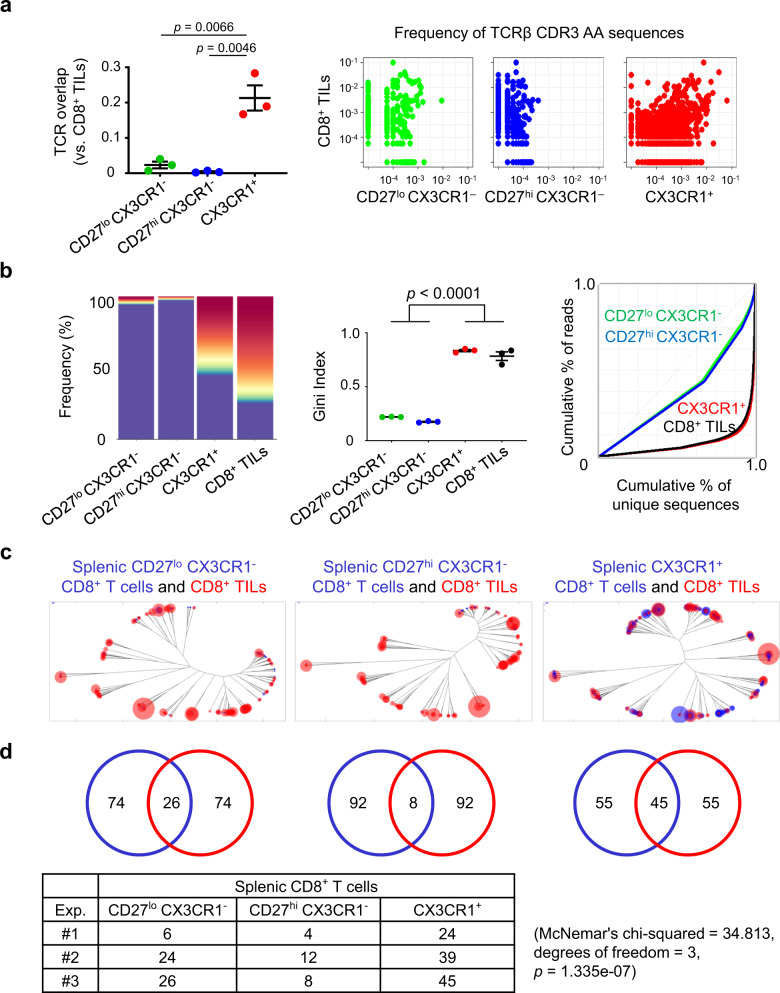
Effective ICI therapy induces a high degree of TCR sequence similarity and clonality between tumor-infiltrating CD8^+^ T cells and peripheral CX3CR1^+^ CD8^+^ T cells. **a**–**d** MC38 tumor-bearing mice were treated with anti-CTLA-4 antibody (Ab) and anti-PD-L1 Ab. Three subsets of splenic CD8^+^ T cells determined by CD27 and CX3CR1 expression (CD27^lo^ CX3CR1^−^, CD27^hi^ CX3CR1^−^, and CX3CR1^+^), and CD8^+^ tumor-infiltrating lymphocytes (TILs) were isolated 2 weeks after the initiation of the treatment for TCR repertoire and clonality analysis. **a** TCR repertoire overlap by Morisita’s index (left) and representative pairwise scatter plots of the frequency of TCRβ CDR3 amino acid (AA) sequences between each subset of splenic CD8^+^ T cells and CD8^+^ TILs (right). *n* = 3 independent experiments. **b** TCR clonality analysis of three subsets of splenic CD8^+^ T cells and CD8^+^ TILs by top sequence plot (left), Gini index (center), and Lorenz curve (right). The most abundant 100 AA sequences are colored while other less frequent clones are in purple in the top sequence plot. *n* = 3 independent experiments for Gini index. The top sequence plot and Lorenz curve are representative of three independent experiments. **c** Representative overlapped weighted TCR repertoire dendrograms by ImmunoMap analysis between three subsets of splenic CD8^+^ T cells (blue) and CD8^+^ TILs (red). The distance of the branch ends represents sequence distance, and the size of circles denotes the frequency of sequence. The data shown are representative of three independent experiments. **d** Number of dominant motifs within top 100 productive sequences shared between three subsets of splenic CD8^+^ T cells (blue) and CD8^+^ TILs (red) in (**c**). The data table shows the number of dominant motifs shared between three subsets of splenic CD8^+^ T cells and CD8^+^ TILs from three independent experiments. One-way repeated-measures ANOVA with Tukey’s multiple comparisons (**a**, **b**). Values are mean ± SEM. Source data are provided as a Source Data file.

The high frequency of tumor-specific CD8^+^ T cells in the CX3CR1^+^ subset before and during ICI treatment is suggestive that heterogeneous tumor-infiltrating CD8^+^ T cells are also enriched in the CX3CR1^+^ subset. To this end, we performed TCR sequencing on isolated CD8^+^ TILs and splenic CD27^lo^ CX3CR1^−^, CD27^hi^ CX3CR1^−^, and CX3CR1^+^ CD8^+^ T cells from MC38 tumor-bearing mice treated with combined anti-CTLA-4/PD-L1 therapy (Supplementary Fig. 5a, b). Comparison of the TCR repertoire in CD8^+^ TILs and three subsets of splenic CD8^+^ T cells demonstrated a high degree of overlap in TCR usage between CD8^+^ TILs and splenic CX3CR1^+^ CD8^+^ T cells (Fig. 4a and Supplementary Fig. 6) as determined by the Morisita’s overlap index^45^.

Next, we evaluated TCR clonality in CD8^+^ TILs and three subsets of splenic CD8^+^ T cells 2 weeks after anti-CTLA-4/PD-L1 therapy (Fig. 4b). The 100 most abundant TCR clones (colored) comprised more than 50% of TCR sequences in the splenic CX3CR1^+^ subset and CD8^+^ TILs while the majority of TCR sequences in the splenic CX3CR1^−^subsets were constituted of less frequent clones (purple). To quantify the skewness of the clonal distribution, we measured the Gini index, and found similarly higher clonality in the splenic CX3CR1^+^ subset and CD8^+^ TILs compared to splenic CX3CR1^−^subsets. Lorenz curves for splenic CX3CR1^+^ subset and CD8^+^ TILs were far from the equidistribution line, suggesting unequal distribution and skewing of the TCR repertoire.

### ICI therapy induces a high degree of TCR sequence similarity and clonality between CD8^+^ TILs and the peripheral CX3CR1^+^ CD8^+^ T cells

TCRs that recognize the same antigen may not be the exact TCR clonotypes but have highly homologous sequences and share similar sequence features^46,47^. Unlike Morisita’s overlap index, a bioinformatics program, ImmunoMap^48^, allows us to analyze biological sequence similarity between peripheral and intratumoral CD8^+^ T cells. The structural clones expanded in the splenic CX3CR1^+^ CD8^+^ T-cell subsets and CD8^+^ TILs were generally shared compared to the CX3CR1^−^subsets and CD8^+^ TILs as visualized by the dendrograms (Fig. 4c and Supplementary Fig. 7a, b) and by tracking dominant motifs (Fig. 4d) in 2 weeks after anti-CTLA-4/PD-L1 therapy.

Analysis of the top six dominant CDR3β amino acid (AA) sequences in splenic CX3CR1^+^ CD8^+^ T cells and CD8^+^ TILs revealed they shared a highly frequent AA sequence (CASSLVGNQDTQYF) in all three independent experiments (Supplementary Table 1, yellow highlighted and Supplementary Data 1). Although the most frequent AA sequence, CASSPRLGDNYAEQFF, in splenic CX3CR1^+^ CD8^+^ T cells was not identified in CD8^+^ TILs in the same experiment (Exp. #1 in Supplementary Table 1a), this AA sequence had a high degree of sequence homology with dominant AA sequences, CASSPGYAEQFF and CASSPGQGYAEQFF in CD8^+^ TILs, located in the same branch of the dendrogram (Supplementary Table 1b, blue highlighted and Supplementary Fig. 7a). Similarly, abundant AA sequences, CASSPGRGYEQYF in splenic CX3CR1^+^ CD8^+^ T cells and CASSSGTYEQYF in CD8^+^ TILs clustered largely in the same branch, indicating that they shared a high degree of similarity (Exp. #2 in Supplementary Table 1, green highlighted and Supplementary Fig. 7b). Collectively, these findings suggest TCR repertoires in peripheral CX3CR1^+^ CD8^+^ T-cell clones reflect the TCR repertoires in CD8^+^ TILs, and CX3CR1 on PB CD8^+^ T cells may act as a dynamic biomarker during the course of effective anti-CTLA-4/PD-L1 therapy.

### Expansion of the CX3CR1^+^ subset in PB CD8^+^ T cells correlates with improved response to anti-PD-1 therapy and survival in patients with NSCLC

We next explored whether changes in the frequency of the CX3CR1^+^ subset in PB CD8^+^ T cells correlate with response to ICI in patients. We evaluated 36 patients with NSCLC treated with anti-PD-1 Ab (pembrolizumab or nivolumab). PB was prospectively obtained at baseline (before treatment initiation) and every 3–6 weeks during therapy for 12 weeks. All patients had pretreatment tumor tissue available to assess PD-L1 expression. When additional tumor tissues were available, the frequency of TILs and TMB was also analyzed as described before^49^. Baseline characteristics of 36 NSCLC patients are described in Supplementary Table 2. Clinical response in individual patients was derived from investigator-reported data per iRECIST criteria^50^ at the 12 week time point. Overall response rates (ORR) which include a complete response (CR) and partial response (PR) were 36.7% and 20% for patients with a PD-L1 tumor proportion score (TPS) of 50% or greater and 1–49%, respectively, in line with previous studies^51,52^.

**Fig. 5:**
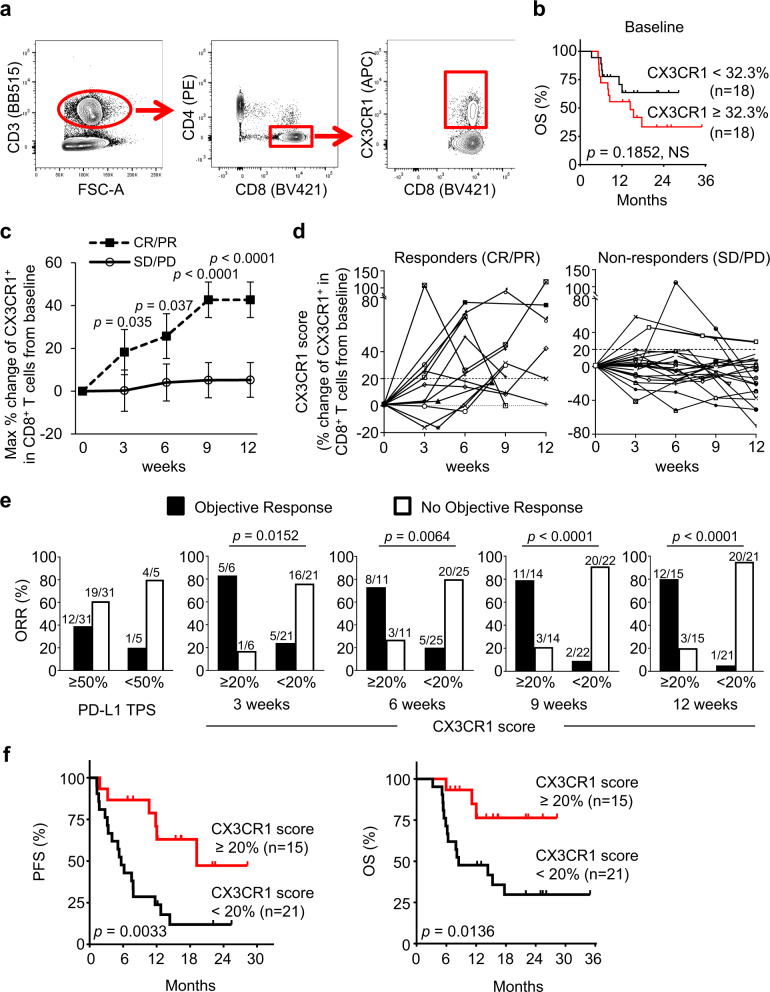
Expansion of the CX3CR1^+^ subset in PB CD8^+^ T cells correlates with response to anti-PD-1 therapy and better survival in patients with NSCLC. **a** Gating strategy for identifying CX3CR1^+^ CD8^+^ T cells in peripheral mononuclear blood cells. Cells were first gated for lymphocytes (SSC-A vs. FSC-A) and for singlets (FSC-H vs. FSC-A). **b** Overall survival (OS) of patients with high (*n* = 18) and low (*n* = 18) pretreatment frequency of the CX3CR1^+^ subset in PB CD8^+^ T cells. Cut-points by median baseline frequency of the CX3CR1^+^ subset in PB CD8^+^ T cells. **c** The largest % change of the CX3CR1^+^ subset in PB CD8^+^ T cells from baseline by the given time point in responders (CR/PR: *n* = 13) and non-responders (SD/PD: *n* = 23) of 36 NSCLC patients treated with anti-PD-1 therapy. CR/PR: complete and partial response, SD/PD: stable and progressive disease. *P-*values were calculated by a two-tailed Mann–Whitney *U*-test. Values are median ± SEM. **d** Percent change of the CX3CR1^+^ subset in PB CD8^+^ T cells from baseline (CX3CR1 score) in responders and non-responders. **e** Objective response rate (ORR) for high and low PD-L1 tumor proportion score (TPS) and PB CX3CR1 score at 3, 6, 9, and 12 weeks. ORR was analyzed by Fisher’s exact test. **f** Progression-free survival (PFS) and OS for high vs. low CX3CR1 score. *P-*values were calculated by a log-rank (Mantel–Cox) test (**b, f**). NS, not significant. Source data are provided as a Source Data file.

We analyzed the frequency of the CX3CR1^+^ subset in PB CD8^+^ T cells from 36 NSCLC patients treated with anti-PD-1 Ab (Fig. 5a). The median baseline frequency of the CX3CR1^+^ subset among CD8^+^ T cells was 32.3% (6.1–76.3%) with no difference of overall survival (OS) between the high- and low-frequency groups (Fig. 5b). Because the pretreatment frequency of CX3CR1^+^ CD8^+^ T cells was variable between patients, to assess the effect of anti-PD-1 therapy on NSCLC patient’s CD8^+^ T cells, we calculated the percent change from baseline in the frequency of the CX3CR1^+^ subset in PB CD8^+^ T cells at all post-treatment time points available (3–12 wk post-treatment initiation). We evaluated the largest change (maximal percent change) of the CX3CR1^+^ subset in PB CD8^+^ T cells from baseline by the given time point in responders and non-responders. The maximal percent change of the CX3CR1^+^ subset was substantially higher in responders than non-responders as early as 3 weeks from the initiation of the treatment (Fig. 5c). Next, we obtained estimates of the area under the curve (AUC) and corresponding 95% confidence interval (CI) using a logistic regression model, and the optimal cutoff score for discriminating between groups using the Youden’s index criterion^53^. These analyses revealed that an increase of CX3CR1^+^ CD8^+^ T-cell subsets by 15.5–21.2% from baseline segregated responders from non-responders at 3–12 weeks associated with higher odds ratio, sensitivity, specificity, positive predictive value (PPV), and negative predictive value (NPV) (Supplementary Table 3 and Supplementary Fig. 8). Hereafter the percent change of the CX3CR1^+^ subset in PB CD8^+^ T cells from baseline are designated as a “CX3CR1 score”. Figure 5d shows longitudinal CD8 T-cell responses in individual patients. We found at least 20% increase of the CX3CR1 score in 92.3% (12/13) of responders compared to only 13.0% (3/23) of non-responders.

Based on these results, we hypothesized that a cutoff of at least 20% increase of the CX3CR1 score would correlate with response to anti-PD-1 therapy and assessed the association between the CX3CR1 score and objective response. The maximal CX3CR1 score of at least 20 started to associate with ORR at 3 weeks (*P* = 0.0152) (odds ratio: 16.0; 95% CI: 1.5–171.2), and became a strong correlate of response at 9 weeks (*P* < 0.0001) (odds ratio: 36.7; 95% CI: 5.3–253.8) (Fig. 5e). Next, we analyzed the corresponding sensitivity, specificity, PPV, and NPV of the CX3CR1 score and the PD-L1 TPS. The maximal CX3CR1 score of at least 20 demonstrated remarkably high PPV, NPV, sensitivity, and specificity, and identified response in 21/27 (77.8%), 28/36 (77.8%), 31/36 (86.1%), and 32/36 (88.9%) at 3, 6, 9, and 12 weeks, respectively, while a PD-L1 TPS of at least 50% had suboptimal PPV and specificity, and correctly identified response only in 16/36 (44.4%) (Table 1). Notably, tumor tissues were available for assessing the frequency of TILs and TMB for only 66.6% (24/36) and 61.1% (22/36) of NSCLC patients, respectively (Supplementary Table 4), suggesting limitation of these analyses, in line with previous reports^54,55^. Lastly, we evaluated the correlation between the CX3CR1 score and survival. The median time of follow-up was 22.3 months (range 3.3–35.5). We found that at least 20% increase of the CX3CR1 score by 12 weeks was associated with better progression-free survival (PFS) (hazard ratio for death or disease progression: 0.28; 95% CI: 0.13–0.62; *P* = 0.0033) and OS (hazard ratio for death, 0.24; 95% CI: 0.09–0.61; *P* = 0.0136) (Fig. 5f). The median PFS and OS among patients with a CX3CR1 score of <20% were 5.7 and 8.6 months, respectively, while the median PFS and OS among patients with a CX3CR1 score of at least 20% by 12 weeks were 19.5 months and not reached.

**Table 1:** Comparison of biomarker performance between PD-L1 TPS and the CX3CR1 score.

	PD-L1 TPS ≥50% (*n* = 36)	Peripheral blood CX3CR1 score ≥20%			
		At 3 weeks (*n* = 27)	At 6 weeks (*n* = 36)	At 9 weeks (*n* = 36)	At 12 weeks (*n* = 36)
PPV	38.7% (12/31)	83.3% (5/6)	72.7% (8/11)	78.6% (11/14)	80.0% (12/15)
NPV	80.0% (4/5)	76.2% (16/21)	80.0% (20/25)	90.9% (20/22)	95.2% (20/21)
Sensitivity	92.3% (12/13)	50.0% (5/10)	61.5% (8/13)	84.6% (11/13)	92.3% (12/13)
Specificity	17.4% (4/23)	94.1% (16/17)	87.0% (20/23)	87.0% (20/23)	87.0% (20/23)
Accuracy	44.4% (16/36)	77.8% (21/27)	77.8% (28/36)	86.1% (31/36)	88.9% (32/36)

CX3CR1 score, the percent change of the CX3CR1^+^ subset in peripheral blood CD8^+^ T cells from baseline.

*PPV* positive predictive value, *NPV* negative predictive value.

The majority of patients (86.1%: 31/36) in our cohort had NSCLC with a PD-L1 TPS of at least 50% and was treated with pembrolizumab, which was approved by the U.S. Food and Drug Administration in 2016. Therefore, we evaluated the relationship between the CX3CR1 score and response to anti-PD-1 therapy in this population. The CX3CR1 score was a correlate of response, and at least 20% increase of the CX3CR1 score by 12 weeks was associated with better clinical outcome by ORR, PFS, and OS (Supplementary Fig. 9a–c). Although the number is small, the CX3CR1 score was a correlate of response in patients with a PD-L1 TPS < 50% (Supplementary Fig. 9d). We also evaluated whether prior treatment with chemotherapy or radiation might have affected the utility of the CX3CR1 score, but did not observe the impact of either prior chemotherapy or radiation on the biomarker performance (Supplementary Fig. 9e). Taken together, the CX3CR1 score is highly correlated with a patient’s clinical response and survival early on-treatment.

Of note, there was no significant difference in the maximal percent change of CD8^+^ T cells in PB CD3^+^ T cells from baseline between responders and non-responders at any time point (Supplementary Table 5 and Supplementary Fig. 10a). Accordingly, changes in the frequency of CD8^+^ T cells among PB CD3^+^ T cells did not correlate with OS and PFS in all NSCLC patients (Supplementary Fig. 10b) and NSCLC patients who had a PD-L1 TPS of at least 50% and were treated with pembrolizumab (Supplementary Fig. 10c).

## Discussion

The lack of robust predictive biomarker for response is a major obstacle of ICI therapy. Blood-based mechanism-driven dynamic biomarkers that reflect constantly evolving TME would be ideal, and intense efforts are ongoing to identify circulating biomarkers for ICI response^4–7,19–23^. Here, we provide evidence in tumor-bearing mice that: (1) effective ICI therapy correlates with the increased frequency and TCR clonality of peripheral CX3CR1^+^ CD8^+^ T cells that identify an enriched repertoire of neoantigen- and TAA-specific CD8^+^ T cells; (2) the frequency of CX3CR1^+^ but not Ki-67^+^ PB CD8^+^ T cells remained elevated during ICI therapy; and (3) there is a high degree of TCR sequence overlap and similarity between CD8^+^ TILs and the peripheral CX3CR1^+^ subset during ICI therapy. Furthermore, analysis of longitudinal PB samples obtained from a cohort of NSCLC patients highlights the potential clinical utility of CX3CR1 as a useful blood-based biomarker to predict response to ICI early after initiation of therapy.

Mechanistically, there are some potential advantages for CX3CR1 as a blood-based biomarker. First, previous studies have shown the correlation of proliferation (Ki-67), co-stimulatory (ICOS), and/or co-inhibitory (PD-1, CTLA-4, and 2B4) markers alone, or in combination with patients responding to ICI therapy^24,25,28,56,57^. However, upregulation of these markers is reversible, and maybe transient on PB T cells^35^ while sustained expression of these markers is observed on TILs. In contrast, differentiation of CX3CR1^int^ to CX3CR1^hi^ subsets is unidirectional^30,31^, and CX3CR1 is irreversibly expressed once T cells are fully differentiated. In agreement with this, we found CX3CR1 but not Ki-67 remains elevated in tumor-specific CD8^+^ T cells during ICI therapy in preclinical models. Additionally, many responding NSCLC patients in our cohort maintained the level of CX3CR1 expression on CD8^+^ T cells above their baseline. Second, given the low levels of CXCR3 expression in CX3CR1^+^ CD8^+^ T cells (Fig. 2b), which is required to traffic to tumors^41^, it is possible that CX3CR1^+^ CD8^+^ T cells remain in circulation, and might not actively traffic to the tumor unless fractalkine (CX3CL1), the ligand of CX3CR1 is produced from the TME. In support with this notion, a recent study showed a higher fraction of CX3CR1^+^ CD8^+^ T cells in PB compared with tumors in NSCLC patients^58^. Furthermore, we have recently reported that adoptively transferred tumor-specific CX3CR1^−^CD8^+^ T cells generate CX3CR1^+^ CD8^+^ T cells upon in vivo stimulation, traffic to the TME, and mediate effective regression of established tumors^43^. In contrast, CX3CR1^+^ CD8^+^ T cells had no impact on established tumors compared with the CX3CR1^−^ subset and became dominant in PB in a preclinical model^43^. These features of CX3CR1^+^ CD8^+^ T cells might have contributed to the greater accuracy of the CX3CR1 score in our cohort and make them uniquely suitable for a circulating T-cell biomarker.

Increased TCR clonality can be identified in tumors and associates with the response to anti-PD-1 therapy^10^; however, the role of PB TCR clonality in predicting response to ICI remains elusive. PB T cells contain highly diverse TCR repertoires, the majority of which are not specific to the tumor; therefore, changes of PB TCR clonality would be difficult to detect even in responders. Identification of markers to detect tumor-specific T cells in PB might overcome this limitation. PD-1 was found to be a cell-surface marker to identify PB neoantigen-reactive T cells^44^, and a recent study showed TCR clonality changes can be observed in PB PD-1^+^CD8^+^ T cells in NSCLC patients treated with anti-PD-1 therapy^59^. Although additional clinical studies are required to determine the predictive and prognostic significance of the TCR clonality in PB CX3CR1^+^ CD8^+^ T cells, our work has also provided insight into the utility of TCR clonality in the peripheral T-cell subset in response to combined CTLA-4/PD-L1 blockade therapy. Our findings of high clonality in peripheral CX3CR1^+^ CD8^+^ T cells that contain intratumoral CD8^+^ T-cell repertoires also align with emerging evidence from high-dimensional profiling of PB T cells by single-cell RNA and/or TCR sequencing showing the expansion of cytotoxic effector memory CD8^+^ T cells that contain novel intratumoral TCR clonotypes in patients responding to ICI therapy^22,60,61^.

A positive correlation of the increased CX3CR1^+^ CD8^+^ T cells and response to anti-CTLA-4 Ab and anti-PD-1 Ab in our preclinical studies warrants further clinical investigation. There is an unmet clinical need in establishing reliable predictive biomarkers for combined anti-PD-1 and anti-CTLA-4 therapy, where the risk of severe immune-related adverse events (irAEs) is as high as the proportion of patients responding to combined ICI^62,63^. Although synergistic antitumor efficacy of anti-CTLA-4 Ab and anti-PD-1 Ab has been observed in a CT26 tumor model^64^, we did not see the difference of tumor control or the frequency of PB CX3CR1^+^ CD8^+^ T cells in mice treated by monotherapy or combined therapy (Fig. 1e, f). The reason for this remains unknown but is likely because we started treatment on day 13 when the tumors were large while the previous study started treatment on day 3^64,65^. Our findings are in line with results from a preclinical study using late-stage tumors^66^.

Our analyses demonstrated that the CX3CR1 score ≥20 at 6–12 weeks could discriminate responders from non-responders with high NPV. One situation where reliable early on-treatment biomarkers with high NPV might be helpful would be for patients with progressive disease at the first imaging assessment (immune-unconfirmed progressive disease^50^). While iRECIST currently requires the confirmation of disease progression in 4–8 weeks^50^, mechanism-driven immune-based biomarker performance can be integrated into the initial assessment of response to immunotherapy, and might enable early identification of patients whose tumors are non-responsive to the initial regimen and who would be candidates for other therapies.

Several limitations are associated with this study. In preclinical models, we evaluated TCR clonality in peripheral CX3CR1^+^ CD8^+^ T cells only in highly-mutated MC38 tumor-bearing mice treated with combined anti-CTLA-4/PD-L1 blockade therapy. Therefore, whether ICI therapy induces a change in the TCR clonality in peripheral CX3CR1^+^ CD8^+^ T cells in mice bearing a lower mutational burden or in mice treated with anti-PD-L1 monotherapy remains an open question. PD-1 was found to be a cell-surface marker to identify PB neoantigen-reactive T cells, but PD-1 was also expressed on self-antigen-reactive T cells^44^. Although neoantigen-specific CD8^+^ T cells and TILs were enriched in the CX3CR1^+^ subset in MC38 tumor-bearing mice treated with ICI therapy, it is possible that this subset also contains self-antigen reactive T cells that may have contributed to the increased clonality. Indeed, an increased clonality in PB CD8^+^ T cells can be seen in patients who were treated by anti-CTLA-4 therapy and developed irAEs^67^.

We have identified a link between the percent change of the CX3CR1^+^ subset in PB CD8^+^ T cells from baseline (CX3CR1 score) and better clinical outcome; however, our study is limited by the lack of validation in independent cohorts. Therefore, a future clinical study with an independent dataset is required to confirm the correlation of at least 20% increase of the CX3CR1 score and clinical outcome. Additionally, the majority of NSCLC patients in our cohort had PD-L1 TPS of at least 50%, and were treated with anti-PD-L1 therapy only. Although analysis of a small number of patients with PD-L1 TPS < 50% showed a correlation of the CX3CR1 score and response to anti-PD-L1 therapy, many patients with NSCLC and PD-L1 TPS < 50% undergo chemo-immunotherapy. Yan et al.^33^ has shown that CX3CR1^+^ CD8^+^ T cells withstand chemotherapy during anti-PD-1 therapy in a few melanoma patients, suggesting the potential utility of CX3CR1 expression on circulating CD8^+^ T cells. Further study is needed to determine the utility of the CX3CR1 score in patients undergoing chemo-immunotherapy.

The CX3CR1 score might be convenient to use in a clinical setting from a technical and analytical perspective. While we isolated peripheral blood mononuclear cells (PBMCs) in this study, the same analysis can be done with as little as 1 ml of whole blood without isolating PBMC by density gradient centrifugation. Unlike intracellular proteins, there is no need for a fixation/permeabilization procedure to stain CX3CR1. The CX3CR1^+^ subset can be easily distinguishable from the CX3CR1^−^ subsets in PB CD8^+^ T cells unlike other markers such as PD-1, where the boundary between positive and negative populations might be difficult to set^44^. The CX3CR1 score can be obtained by traditional fluorescence flow-cytometric analysis, and does not require a next-generation sequencing platform, complex algorithm, or bioinformatics analysis. Thus, the results can be readily available for prompt feedback to oncologists and patients.

Tumor PD-L1 expression predicts the likelihood of response to anti-PD-1 therapy and is used as a companion diagnostic for NSCLC but not for several other malignancies such as melanoma. Therefore, it remains elusive whether the CX3CR1 score would be useful in patients with other types of cancer. A recent study showed T-cell invigoration to tumor burden ratio was associated with response to anti-PD-1 Ab and clinical outcome in melanoma patients^24^. Although it was not within the scope of our study, one future area of investigation would be to compare the utility of different PB T-cell biomarkers in the same disease, or evaluate whether combining multiple biomarkers could improve predictive and prognostic values.

In summary, our findings demonstrate that circulating T-cell CX3CR1 reflects dynamic change of CD8^+^ TILs in the tumor microenvironment early after initiation of ICI therapy, and that an increase in the frequency of the CX3CR1^+^ subset in PB CD8^+^ T cells correlates with response to anti-PD-1 therapy early on-treatment in NSCLC patients. Our study provides a rationale for further investigation to test the utility of the circulating T-cell differentiation marker for a wide variety of malignancies in larger prospective trials.

## Methods

### Mice

Male and female C57BL/6 mice and female Balb/c mice were purchased from the Jackson Laboratories. All mice were age matched (7–10 wk old) at the beginning of each experiment and kept under specific pathogen-free conditions and housed in the Laboratory Animal Resources. All animal studies were conducted in accordance with and approved by the Institutional Animal Care and Use Committee (IACUC) at Roswell Park Comprehensive Cancer Center.

### Cell lines

MC38 and CT26 murine colon adenocarcinoma cell lines were gifts from Dr. Weiping Zou (University of Michigan) and Dr. Sharon Evans (Roswell Park Comprehensive Cancer Center), respectively. The murine B16F10 (B16) melanoma cell line was purchased from ATCC. MC38, CT26, and B16 cells were cultured in RPMI (Gibco) supplemented with 10% FBS (Sigma), 1% NEAA (Gibco), 2 mM GlutaMAX-1 (Gibco), 100 U/ml penicillin-streptomycin (Gibco), and 55 μM 2-mercaptoethanol (Gibco). Cells were authenticated by morphology, phenotype, and growth, and routinely screened for *Mycoplasma* by polymerase chain reaction (PCR), and were maintained at 37 °C in a humidified 5% CO_2_ atmosphere.

### In vivo mouse studies

Male or female C57BL/6 mice and female Balb/c mice were inoculated with 5–8 × 10^5^ MC38, CT26 or B16 per mouse on the right flank by subcutaneous injection on day 0. When tumor volume reached ~50 mm^3^, 200 µg of anti-PD-L1 Ab (clone 10 F.9G2, BioXcell), and/or 100 µg of anti-CTLA-4 Ab (clone 9H10, BioXcell) were administered intraperitoneally every 3 days and every other day, respectively. Polyclonal Syrian hamster IgG (BioXcell) and rat IgG2b, κ (BioXcell) were used as isotype control Abs. Tumor volumes were calculated by determining the length of short (*l*) and long (*L*) diameters (volume = *l*^2^ × *L*/2). Experimental endpoints were reached when tumors exceeded 20 mm in diameter or when mice became moribund and showed signs of lateral recumbency, cachexia, lack of response to noxious stimuli, or observable weight loss.

### Single-cell preparations

Blood, spleens, and tumors were harvested from MC38, CT26, or B16 tumor-bearing mice. Spleens were homogenized by forcing the tissue through a cell strainer (70 μm; BD Biosciences). Red blood cells in the blood and spleen were lysed using ACK Lysis Buffer (Gibco). Tumors were cut into small pieces of 2–4 mm. Single-cell suspensions were obtained by mechanical dispersion consisting of two 30-min incubations at 37 °C, 5% CO_2_ in 5 ml RPMI 1640 (Gibco) and tumor dissociation kit (Miltenyi Biotec) in C Tubes (Miltenyi Biotec) interspersed with three mechanical dispersions on a GentleMACS dissociator (Miltenyi Biotec). The tumor cell suspensions were then filtered through a cell strainer (70 μm; BD Biosciences).

### Flow cytometry and cell sorting

Antibodies used in this study are commercially available and listed in Supplementary Table 6. Single-cell suspensions of mouse blood, spleens, and tumors were prepared as above for flow-cytometric analysis. Murine cells were incubated with antibodies in PBS containing 2% FBS for 20 min at room temperature after a blockade by anti-CD16/CD32 (BD Biosciences). Live/dead cell discrimination was performed using Live/Dead Fixable Aqua Dead Cell Stain Kit or LIVE/DEAD Fixable Near-IR Dead Cell Stain Kit (Life Technologies). We used the tetramer staining assay with a peptide-MHC tetramer tagged with PE (H-2D^b^-restricted ASMTNMELM for MC38-bearing mice and H-2Ld-restricted SPSYVYHQF for CT26-bearing mice (The NIH Tetramer Core Facility)) to analyze the percentages of tumor antigen-specific CD8^+^ T cells. For intracellular staining, surface-stained cells were fixed and permeabilized using a Foxp3 fixation/permeabilization kit (eBioscience) or Fixation/Permeabilization Solution Kit (BD Biosciences) according to the manufacturer’s recommendations. For phenotypic analysis of PB T cells, fresh or cryopreserved PBMC samples were stained with a master mix of antibodies for surface stains including CD3, CD4, CD8, and CX3CR1 after Fc block with human IgG (Sigma) at 12 mg/ml. Samples were acquired using LSR II (BD Biosciences) LSRFortessa (BD Biosciences), or BD FACSARIA II Cell sorter with BD FACSDiva software v8.0 (BD Biosciences) or MA900 Multi-Application Cell Sorter (Sony Biotechnology Inc.) and data analyzed with FlowJo software v10.1.5 (TreeStar).

For TCR sequencing of murine splenic CD8^+^ T cells, single-cell suspensions from freshly isolated splenocytes were stained. CD45^+^ CD3^+^ CD8^+^ T cells were gated, and CD27^lo^ CX3CR1^−^, CD27^hi^ CX3CR1^−^, and CX3CR1^+^ CD8^+^ T cells were sorted using BD FACSAria II Cell Sorter. An EasySep Mouse CD8a Positive Selection Kit II (STEMCELL Technologies) was used to isolate murine CD8^+^ TILs for TCR sequencing.

### DNA isolation, TCRβ CDR3 region sequencing, and repertoire analysis

DNA from flow-isolated murine splenic CD8^+^ T cells and CD8^+^ TILs, and PB CD8^+^ T cells was extracted using QIAamp DNA Micro Kit (QIAGEN). DNA was quantified using Qubit dsDNA BR Assay (Invitrogen). Amplification and sequencing of TCRβ CDR3 regions were performed using ImmunoSEQ immune profiling system at the survey level (Adaptive Biotechnologies)^68^. Sequencing was performed on an Illumina NextSeq system using a 150 cycles mid-output kit (Illumina Inc.). Processed data were uploaded to the ImmunoSEQ Platform (Adaptive Biotechnologies) for bioinformatics analysis. Processed data were downloaded and frequencies/counts for TCR clonotypes and diversity were examined by nucleotide sequences after non-productive reads were filtered out.

T-cell repertoires, comprising all detected CDR3 sequences with annotated V and J gene segment identifications were downloaded directly to the ImmunoSEQ Analyzer from Adaptive Biotechnologies. Metrics of the complete TCR repertoire in each sample, including the number of productive rearrangements, productive clonality, and clonal frequencies were determined using the ImmunoSEQ Analyzer software and confirmed using the LymphoSeq package^69^. All other analyses were performed using the LymphoSeq package and custom scripts in the R statistical software environment. The dissimilarity between sample repertoires was calculated using the Morisita’s Index^45^ by the ImmunoSEQ Analyzer. TCR clonality was calculated as 1-Pielou’s evenness^70^ using the ImmunoSEQ Analyzer. Clonality values approaching 1 indicate a very skewed distribution of frequencies, whereas values approaching 0 indicate that every rearrangement is present at a nearly identical frequency.

TCR repertoires were visualized as weighted dendrograms using ImmunoMap^48^. Only productive top 100 unique CDR3 sequences in the tumor and spleen were considered for analysis. Sequence distances were calculated based on sequence alignment scores using a PAM10 scoring matrix and a gap penalty of 30. Circles are overlaid at the end of the branches corresponding to the CDR3 sequences with diameters proportional to the frequency of the sequences observed in the samples. The dominant motifs shared by each splenic CD8^+^ T-cell subset and CD8^+^ TILs in Fig. 4d are based on the # of exactly matched TCR clonotypes.

### Data reporting

For the clinical study, no statistical methods were used to predetermine sample size. The clinical samples are selected based on availability during the study window. The observed sample size provides a sufficient pool of both responders and non-responders, and produces performance measures (i.e., AUC, sensitivity, etc.) with adequate levels of precision. Randomization was not appropriate or feasible due to the nature of this study. We examined the association between a biomarker measured over time and response. The study followed the Reporting Recommendations for Tumor Marker Prognostic Studies (REMARK)^71^.

### Study design, patients, and specimen collection

The study population consisted of patients who were treated with anti-PD-1 Ab (pembrolizumab or nivolumab) for stages II–IV NSCLC at the Roswell Park Comprehensive Cancer Center. Participants in this study were prospectively recruited for blood sampling between September 2017 and January 2020. Patients who were older than 18 years were eligible, and were consented to the collection and storage of blood samples, the analysis of archived tumor tissue, and the review of their medical records (I 188310), in accordance with the Institutional Review Board of Roswell Park Comprehensive Cancer Center. Peripheral blood was obtained in EDTA-containing tubes before treatment and before each infusion and every 3–6 weeks for 12 weeks. PBMCs were isolated using Lymphocyte Separation Medium (Corning) density gradient centrifugation and stored using standard protocols. Thirty-six patients with PD-L1 IHC positive NSCLC who underwent at least one full dose of anti-PD-1 Ab, blood collection at baseline and post-treatment, and imaging studies were evaluated (Supplemental Table 2).

### Assessment of response

Clinical response to anti-PD-1 therapy was determined as the best response based on immune-related RECIST (iRECIST)^50^ at the 12-week time point, and classified as complete response (CR) and partial response (PR) for responders or stable disease (SD) and progressive disease (PD) for non-responders. Objective responses were confirmed by at least one sequential tumor assessment, and objective response rates were calculated as [(CR + PR) ÷ number of patients] × 100. Fisher’s exact test was used to assess the association between PD-L1 expression or the CX3CR1 score and objective response.

### Immunohistochemical studies

The expression of PD-L1 on the surface of tumor cells and the frequency of CD8^+^ T cells were evaluated by clinical comprehensive immune profiling performed by OmniSeq, Inc.^49^. Briefly, the expression of PD-L1 on the surface of tumor cells was assessed by means of the Dako Omnis platform (Agilent) with the 22C3 pharmDx antibody and scored by published guidelines^72^. Serially sectioned tissue was evaluated for lymphocyte infiltration using the anti-CD8 antibody (clone C8/144B) (Agilent) and assigned a qualitative score of non-infiltrated, infiltrated, or excluded. Non-infiltrated referred to a sparse number of CD8^+^ T cells that infiltrate nests of neoplastic cells and with <5% of the tumor showing an infiltrating pattern. Infiltrated represents frequent CD8^+^ T cells that infiltrate nests of neoplastic cells in an overlapping fashion at least focally and in more than 5% of the tumor. Excluded represents a restriction of more than 95% of all CD8^+^ T cells in a tumor to the periphery or interstitial stromal areas and not actively invading nest or groups of neoplastic cells.

### Tumor mutational burden profiling

Tumor mutational burden (TMB) was evaluated by clinical comprehensive immune profiling performed by OmniSeq, Inc.^49^. In brief, DNA was extracted from each sample and processed for whole-exon DNAseq. TMB was assessed by targeted capture and sequencing of 409 cancer-related genes and amplicon sequencing of 394 immune transcripts, respectively, comprising 1.4 Mb of DNA on samples that met validated quality control (QC) thresholds. Somatic mutation calling was conducted using Ion Torrent Suite software’s variant caller plugin. Mutational burden (MuB) cutoff was derived from a reference population whereby the median MuB was determined on regular basis. This value was used as a baseline and a high MuB was defined as 2× this median value, or a value of 10.0.

### Statistics

The marker expression was summarized by time point and objective response using the mean and standard deviation. Comparisons were made using the Mann–Whitney *U* test. The maximal % change of CX3CR1^+^ T cells was calculated as the largest change (maximal percent change) of the CX3CR1^+^ subset in PB CD8^+^ T cells from baseline by the given time point. The association between the maximal percent change in CX3CR1 or CD8 at pre-specified time points and the objective response was evaluated using a logistic regression model, from which ROC curves and the corresponding AUC were estimated. Confidence intervals were obtained for the AUC using DeLong’s method^73^. The Youden’s index criterion^53^ was used to obtain the optimal cut-point, and performance was evaluated using the corresponding sensitivity, specificity, positive predictive value, and negative predictive value; with 95% confidence intervals obtained using Jeffrey’s prior method. All analyses were conducted in SAS v9.4 (Cary, NC) at a significance level of 0.05.

PFS and OS were defined as the time from the first dose of anti-PD-1 Ab to progression or death (for PFS) or death alone (for OS). The Kaplan–Meier method was used to estimate overall and progression-free survival. Data for patients who were alive or lost to follow-up were censored for OS at the time they were last known to be alive. Data for patients who were alive and did not have disease progression or who were lost to follow-up were censored for the analysis of PFS at the time of the last imaging assessment. This report is based on the final analysis of OS and PFS in NSCLC patients treated with anti-PD-1 therapy, as of the database lock of August 4, 2020.

We used two-tailed Student *t*-test or Mann–Whitney *U*-test for comparisons between two groups, 1-way repeated-measures ANOVA with Turkey-adjusted multiple comparisons or Kruskal–Wallis with Dunn’s multiple comparisons for comparisons more than two groups, or the Mantel–Cox method (log-rank test) for survival analysis using GraphPad Prism 8.02 (GraphPad Software) and the R statistical software. TCR repertoire analysis was performed using ImmunoSEQ software (Adaptive Biotechnologies) and ImmunoMap. *P* < 0.05 was considered statistically significant. Data are presented as mean ± SEM except for Fig. 5c and Supplementary Fig. 10a (median ± SEM).

### Reporting summary

Further information on research design is available in the Nature Research Reporting Summary linked to this article.

## Supplementary information

Supplementary Information

Description of Additional Supplementary Files

Supplementary Data 1

Reporting Summary

## Data Availability

The TCR-seq data were deposited in the National Center for Biotechnology Information Gene Expression Omnibus (NCBI-GEO) under accession number GSE165383, and on Zenodo. The patients did not give their consent for the public availability of their raw sequencing data. DNA sequencing data from lung cancer patients are available with a data share agreement, and can be requested from F.I. (fumito.ito@roswellpark.org). Any other relevant data are available from the corresponding author upon reasonable request. Source data are provided with this paper.

## Source data

Source Data
