# Supplementary material for: T-cell CX3CR1 expression as a dynamic blood-based biomarker of response to immune checkpoint inhibitors
Source: Nat Commun. 2021 Mar 3;12:1402. doi: 10.1038/s41467-021-21619-0 (PMC7930182; doi:10.1038/s41467-021-21619-0)
Supplement: Supplementary file 1 — Supplementary Information [file 41467_2021_21619_MOESM1_ESM.pdf]

T-cell CX3CR1 expression as a dynamic blood-based biomarker of response to immune checkpoint inhibitors

Yamauchi *et al.*

Supplementary Information

Supplementary Figures 1-10
Supplementary Tables 1-6

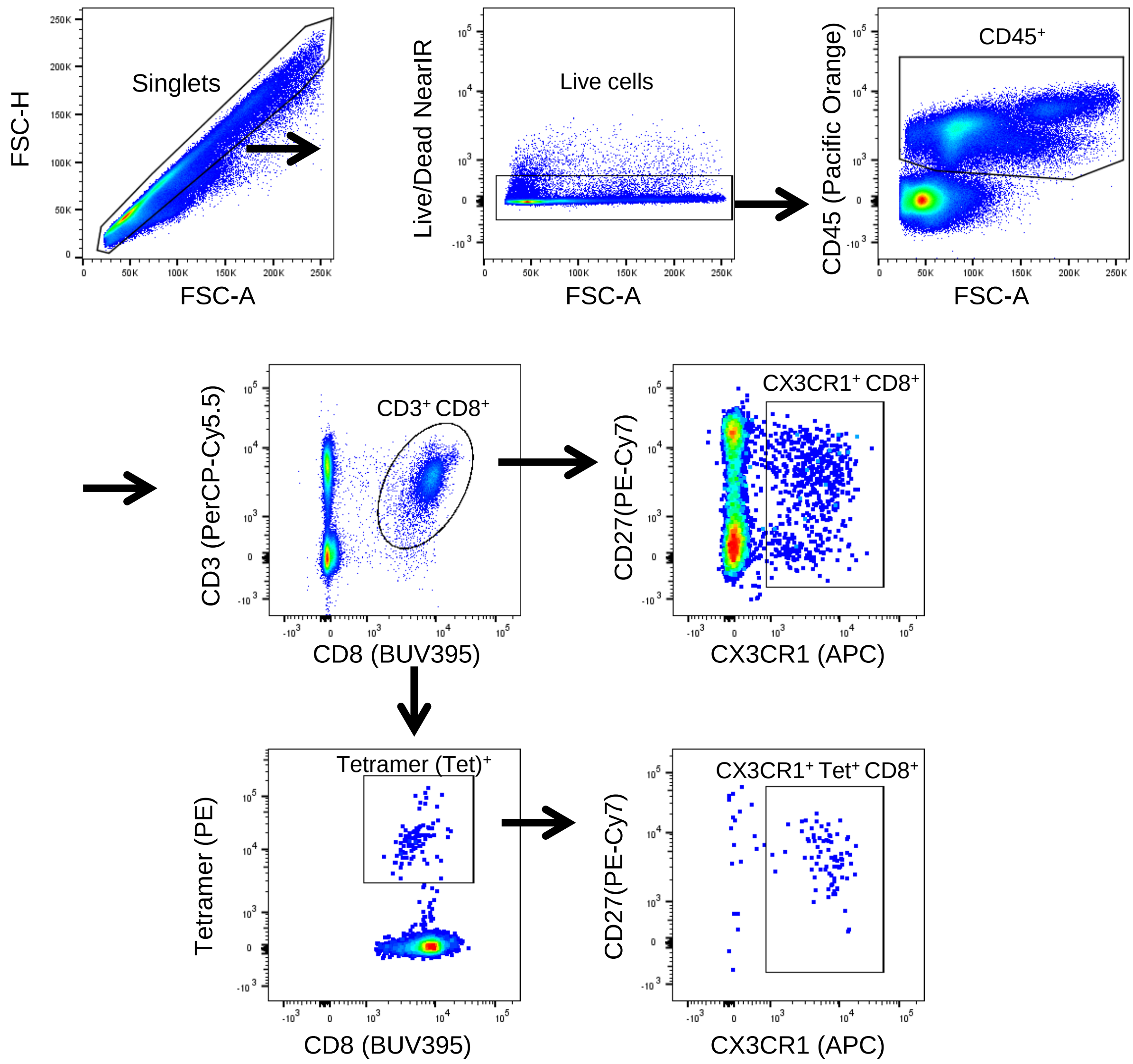

Supplementary Figure 1

Gating strategy for evaluating CX3CR1 and CD27 expression in peripheral CD8^{+} and tetramer (Tet)$^{+}$ CD8^{+} T cells in mice. Peripheral blood (PB) or splenocytes were first gated for lymphocytes (SSC-A vs. FSC-A), and for singlets (FSC-H vs. FSC-A). The singles gate was further analyzed for their uptake of the LIVE/DEAD Fixable Near-IR Dead Cell stain to determine live versus dead cells, and their expression of CD8 (FITC or BUV395) and CD3 or CD90.2 (PerCP-Cy5.5) to identify CD8^{+} T cells. A peptide-MHC tetramer tagged with PE (H-2D^{b}-restricted ASMTNMELM for MC38-bearing mice and H-2Ld-restricted SPSYVYHQF for CT26-bearing mice) was used to analyze the percentages of tumor antigen-specific CD8^{+} T cells. This gating strategy was used in Figures 1, 3, 4, and Supplementary Figures 2 - 5

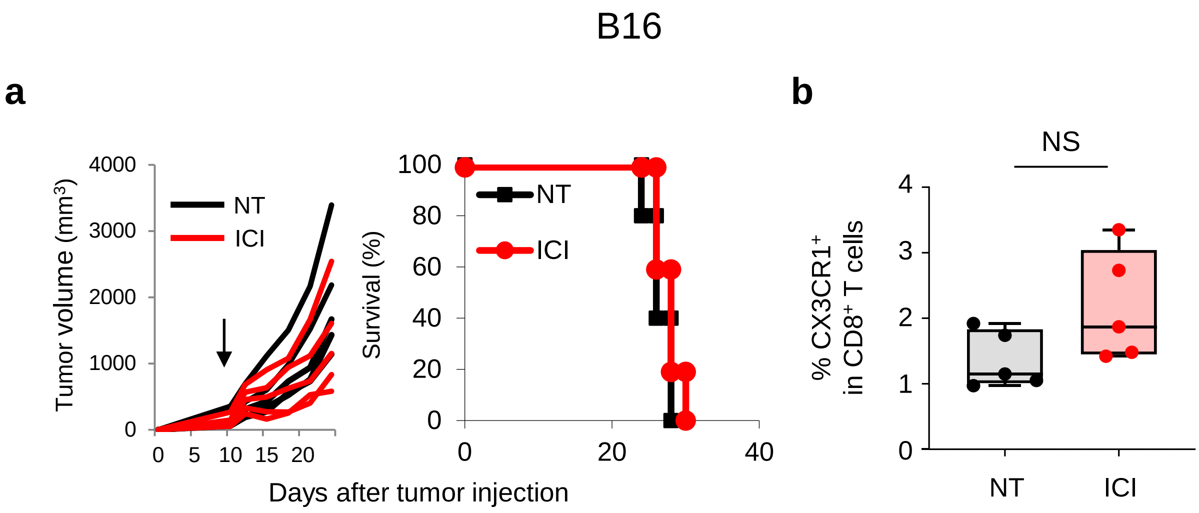

Supplementary Figure 2, Related to Figure 1.

a Individual tumor growth and survival curves in B16 tumor-bearing mice treated with isotype antibody (Ab) (NT) or anti-PD-L1 Ab and anti-CTLA-4 Ab (ICI: Immune checkpoint inhibitors). Arrow indicates initiation of treatment.

b Frequency of CX3CR1^{+} cells among CD8^{+} T cells. Gating strategy: All cells > Size (lymphocytes) > Singlets > Live > CD3^{+} > CD4^{-} CD8^{+}. PB was harvested 2 weeks after initiation of the treatment. NS, not significant by a two-tailed Mann-Whitney *U*-test. Data are presented as mean ± SEM. For **a** and **b**, n = 5 mice in all groups. Data shown are representative of two independent experiments. Box plots: dot, single PB; hinges, 25th and 75th percentiles; middle line, median; whiskers, minimum to maximum value. Source data are provided as a Source Data file.

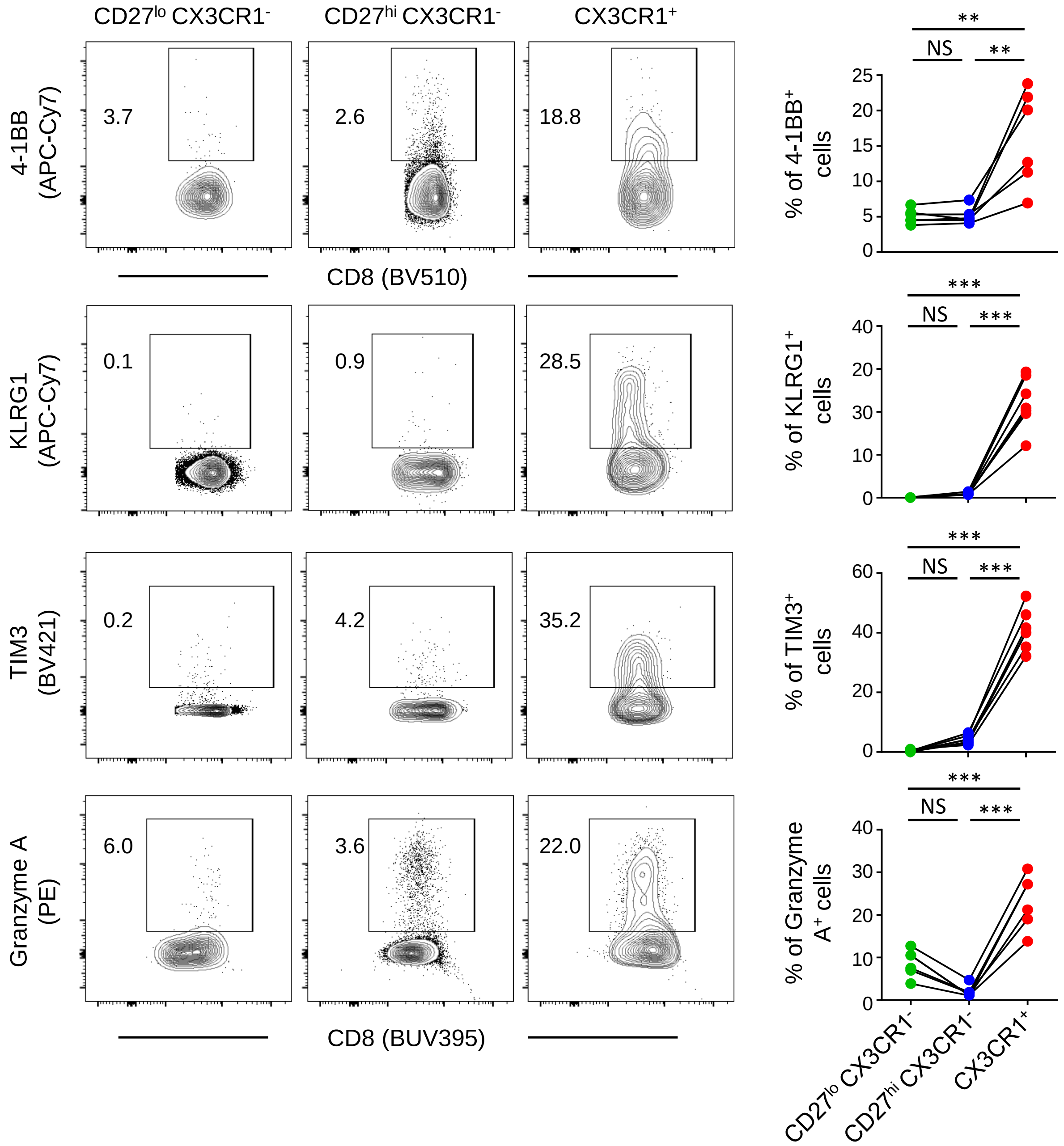

Supplementary Figure 3, Related to Figure 2.

Mice bearing 20-day established MC38 tumors were treated with anti-PD-L1 antibody (Ab) every 3 days. Spleens were harvested 2 weeks after initiation of the treatment. Representative flow-cytometric plots of three subsets (CD27^{lo} CX3CR1^{-}, CD27^{hi} CX3CR1^{-}, and CX3CR1^{+}) of splenic CD8^{+} T cells gated with the single live CD90.2^{+} CD8^{+} population are shown. Data panels show frequency among CD8^{+} T cells. NS, not significant, ***P* < 0.005, ****P* < 0.0001, by one-way repeated measures ANOVA with Tukey's multiple comparisons. n = 6 mice in 4-1BB and granzyme A, n = 7 mice in KLRG1 and TIM3. Data shown are representative of two independent experiments. Source data are provided as a Source Data file.

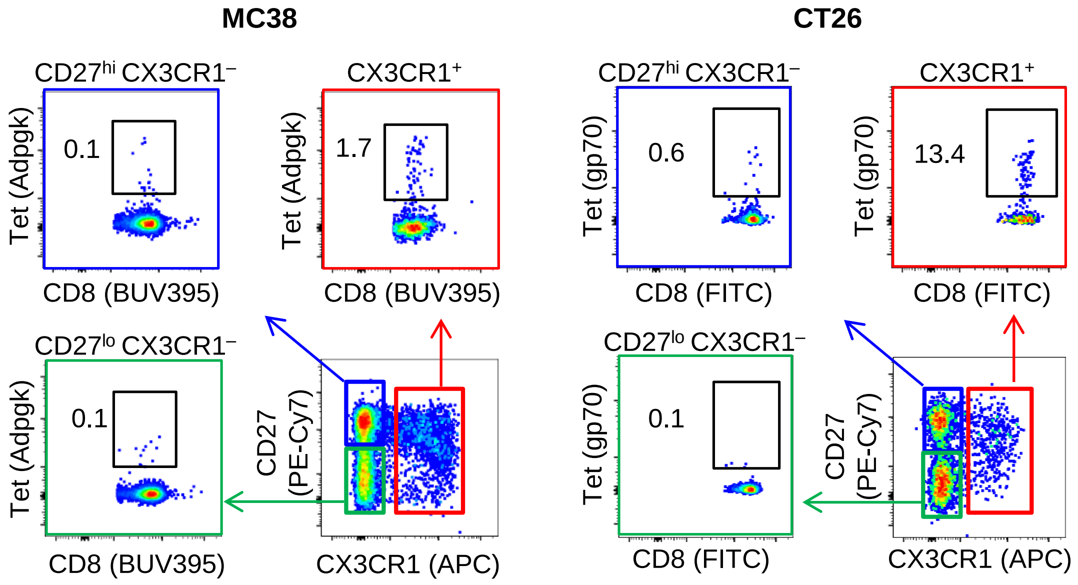

Supplementary Figure 4, Related to Figure 3.

Representative flow cytometric plots showing the frequency of tetramer (Tet)$^{+}$ CD8^{+} T cells in the CD27^{lo} CX3CR1^{-} (green), CD27^{hi} CX3CR1^{-} (blue), and CX3CR1^{+} (red) subsets in peripheral blood (PB) of MC38 (left) or CT26 (right) tumor-bearing mice treated with immune checkpoint inhibitors (ICI; anti-PD-L1 Ab and anti-CTLA-4 Ab) for 2 weeks. Numbers denote percent Tet^{+} cells among CD8^{+} T cells. Two different PE-conjugated tetramers were used to detect CD8^{+} T cells specific for mutated Adpgk protein (Adpgk^{Mut}) in MC38 or shared tumor-associated antigen (TAA), gp70 in CT26 tumor models. Data shown are representative of two independent experiments.

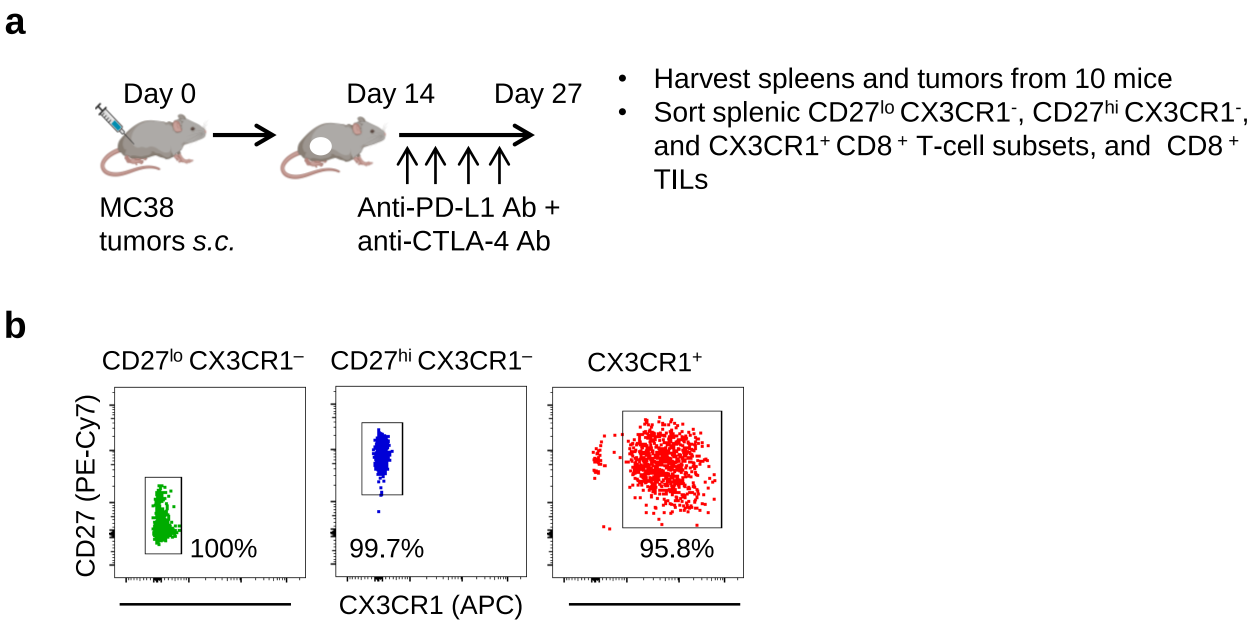

Supplementary Figure 5, Related to Figure 4.

a Experimental scheme of treatment with immune checkpoint inhibitors (ICI), and isolation of three subsets of splenic $CD8^+$ T cells and $CD8^+$ TILs for T-cell receptor (TCR) repertoire and clonality analysis for Figure 4, Supplementary Figures 5b, 6, 7, and Supplementary Table 1.

b Representative flow cytometric plots showing the frequency of splenic $CD27^{lo}$ $CX3CR1^-$ (green), $CD27^{hi}$ $CX3CR1^-$ (blue), and $CX3CR1^+$ (red) $CD8^+$ T cells after flow-sort. Gating strategy: All cells>Size (lymphocytes) > Singlets > Live > $CD45^+$ > $CD3^+$ $CD8^+$ > CD27, CX3CR1.

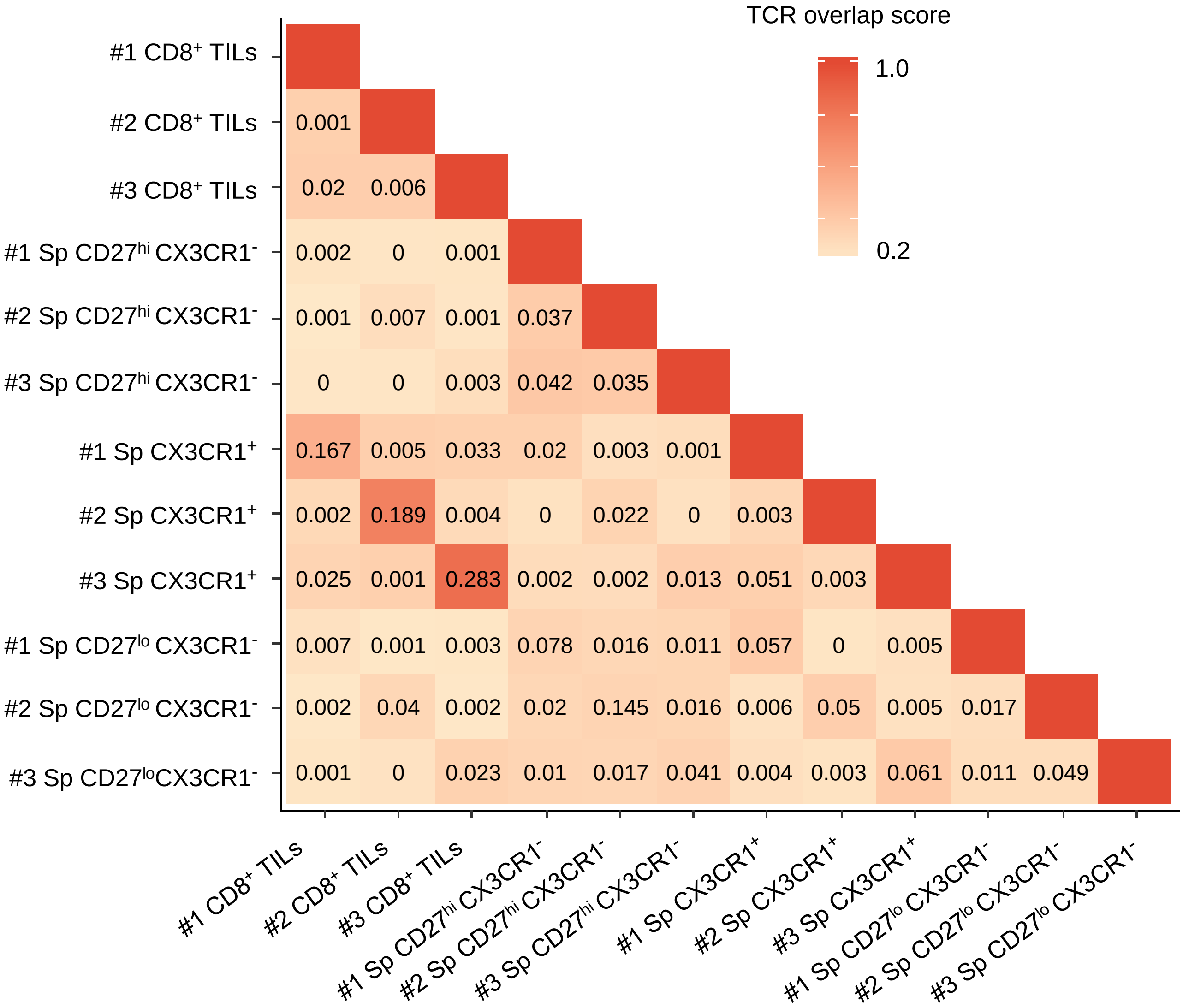

Supplementary Figure 6, Related to Figure 4a.

Pairwise TCRβ repertoire overlaps among different samples from three independent experiments (#1 - #3). Numbers denote TCR repertoire overlaps by Morisita's index. (Sp: spleen, TILs: tumor-infiltrating lymphocytes)

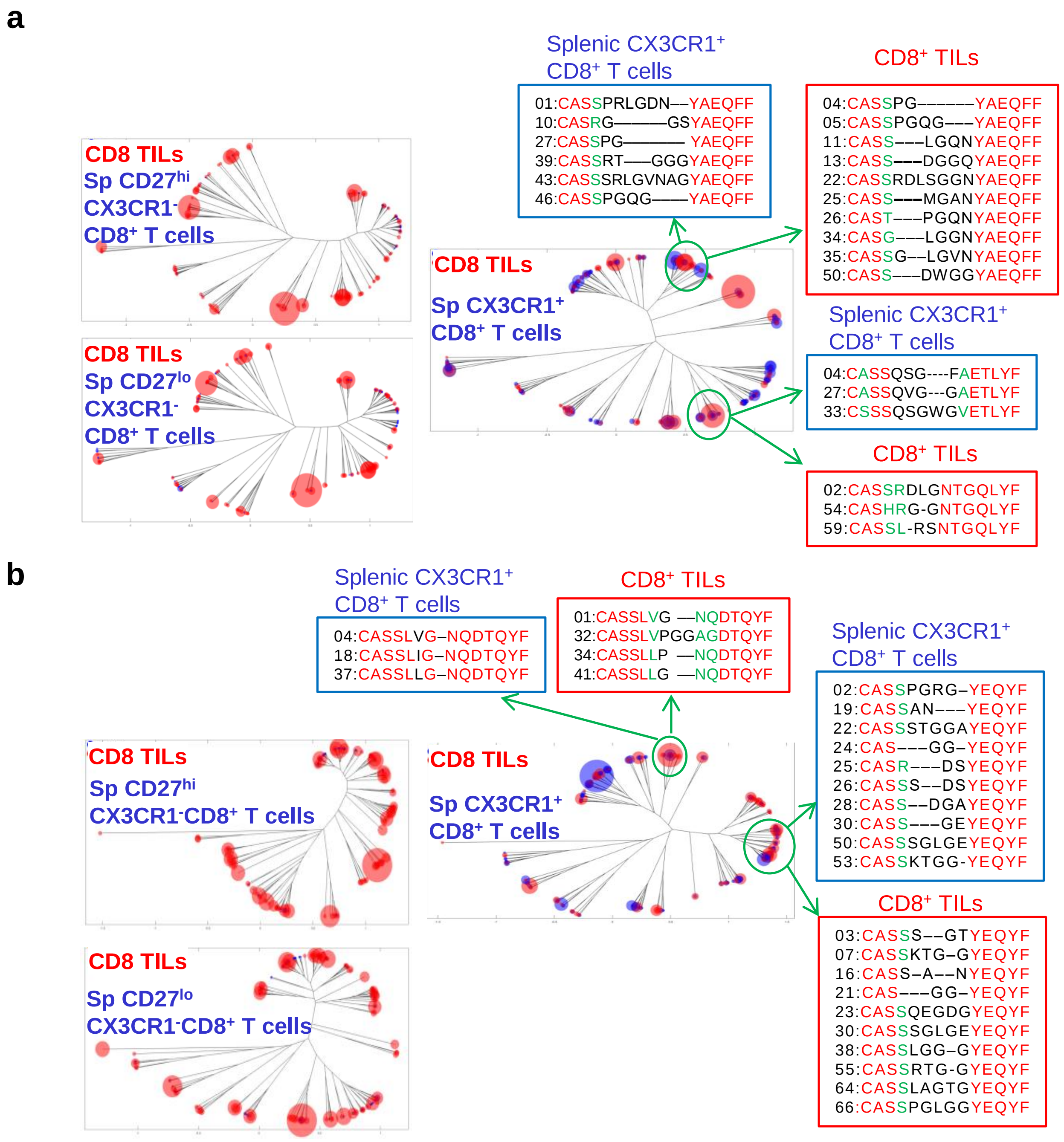

Supplementary Figure 7, Related to Figure 4c and Supplementary Table 1. Representative overlapped weighted TCR repertoire dendrograms by ImmunoMap analysis between three subsets of splenic (Sp) CD8+ T cells (blue) and CD8+ TILs (red) from two independent experiments (**a** and **b**). Dominant motif analysis clusters homologous sequences and selects for clusters contributing to significant proportion of the response. Two dominant motifs are shown representing highly represented structural motifs in each experiment. The numbers denote the ranking of the frequency within the subset. (red = fully conserved amino acids (AA), green = semi-conserved AA, black = non-conserved AA)

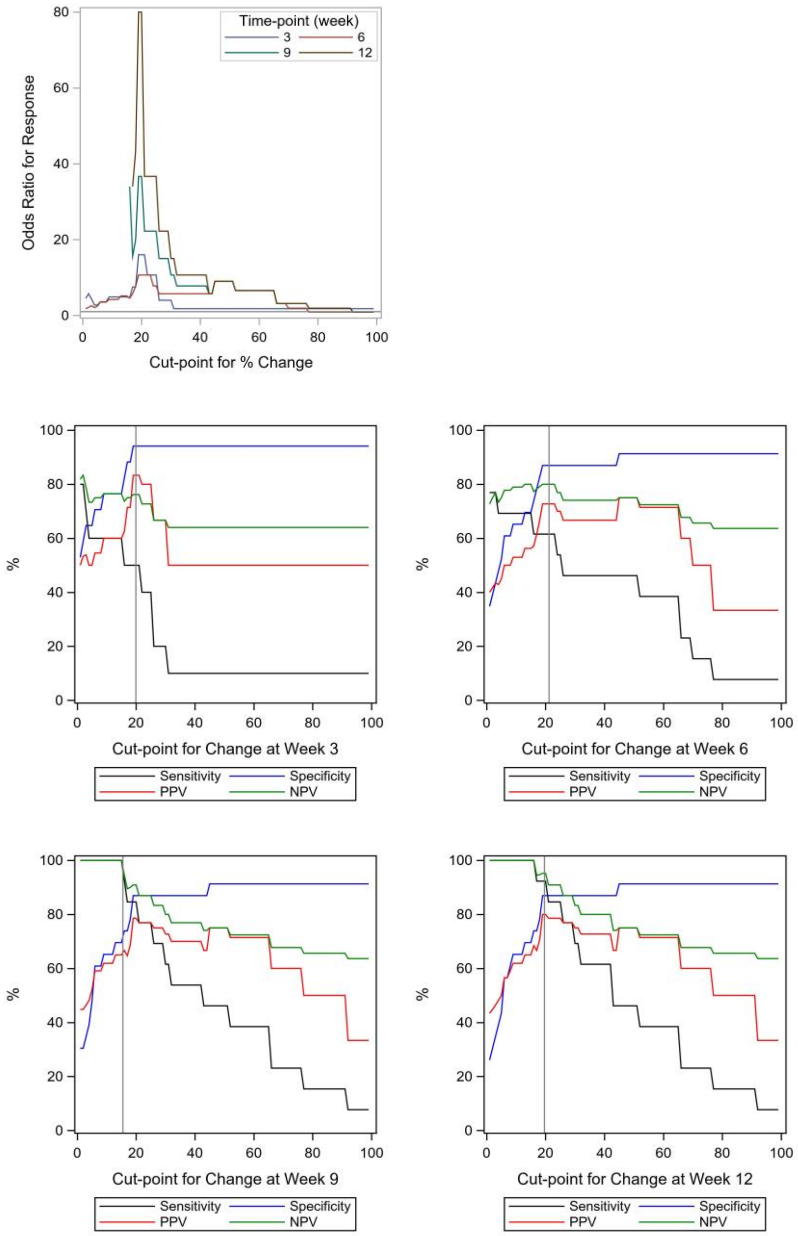

Supplemental Figure 8, Related to Figure 5.

Identification of optimal cut-off score for differentiating responders from non-responders in non-small cell lung cancer (NSCLC) patients (n=36) treated with anti-PD-1 therapy. The largest change (maximal percent change) of the CX3CR1^{+} subset in circulating CD8^{+} T cells from baseline was calculated at 3, 6, 9, and 12 weeks from the initiation of the anti-PD-1 therapy in responders (n=13) and non-responders (n=23). The full range of different cut-offs were tested for odds ratio, sensitivity, specificity, positive predictive value (PPV) and negative predictive value (NPV).

a

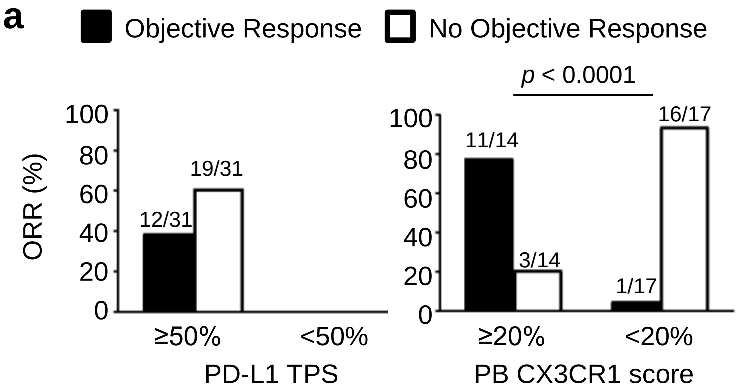

b

	PD-L1 TPS ≥50% (n=31)	CX3CR1 score ≥20% (n=31)
PPV	38.7% (12/31)	78.6% (11/14)
NPV	N/A	94.1% (16/17)
Sensitivity	N/A	91.7% (11/12)
Specificity	N/A	84.2% (16/19)
Accuracy	38.7% (12/31)	87.1% (27/31)

c

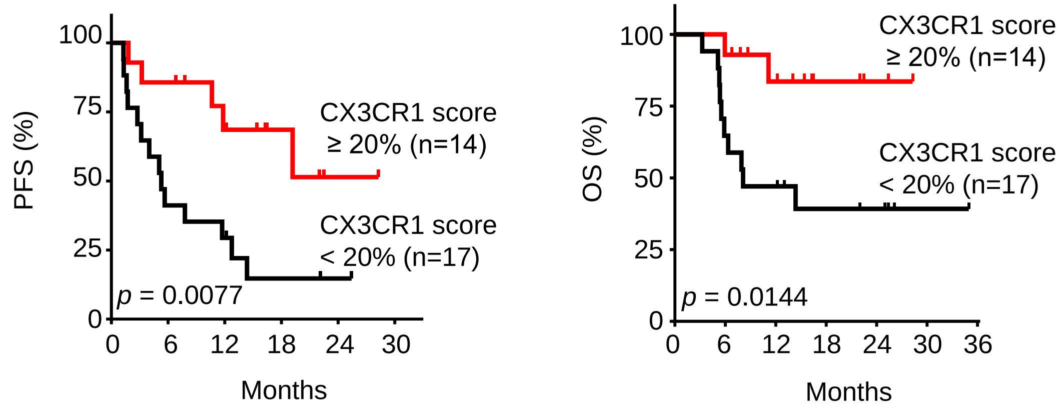

d

Patients with PD-L1 TPS <50%

	PD-L1 TPS ≥50% (n=5)	CX3CR1 score ≥20% (n=5)
PPV	N/A	100% (1/1)
NPV	80% (4/5)	100% (4/4)
Sensitivity	N/A	100% (1/1)
Specificity	100% (4/4)	100% (4/4)
Accuracy	80% (4/5)	100% (5/5)

e

Patients with prior radiation or chemotherapy

	Patients with prior radiation CX3CR1 score ≥20% (n=25)	Patients with prior chemotherapy CX3CR1 score ≥20% (n=9)
PPV	81.8% (9/11)	100% (2/2)
NPV	92.9% (13/14)	100% (7/7)
Sensitivity	90% (9/10)	100% (2/2)
Specificity	86.7% (13/15)	100% (7/7)
Accuracy	88% (22/25)	100% (9/9)

Supplementary Figure 9, Related to Figure 5.

a Objective response rate (ORR) for PD-L1 tumor proportion score (TPS) and PB CX3CR1 score at 12 weeks for non-small cell lung cancer (NSCLC) patients with a PD-L1 TPS > 50% treated with Pembrolizumab. ORR was analyzed by Fisher's exact test.

b Comparison of biomarker performance between PD-L1 TPS and CX3CR1 score at 12 weeks in NSCLC patients with a PD-L1 TPS > 50% treated with Pembrolizumab.

c Kaplan-Meier progression free survival (PFS) and overall survival (OS) for high versus low CX3CR1 score in NSCLC patients with a PD-L1 TPS > 50% treated with Pembrolizumab. *P* values were calculated by a log-rank (Mantel-Cox) test.

d Comparison of biomarker performance between PD-L1 TPS and CX3CR1 score at 12 weeks in patients who had NSCLC with a PD-L1 TPS < 50%.

e Utility of the CX3CR1 score at 12 weeks in NSCLC patients with prior radiation or chemotherapy. Source data are provided as a Source Data file.

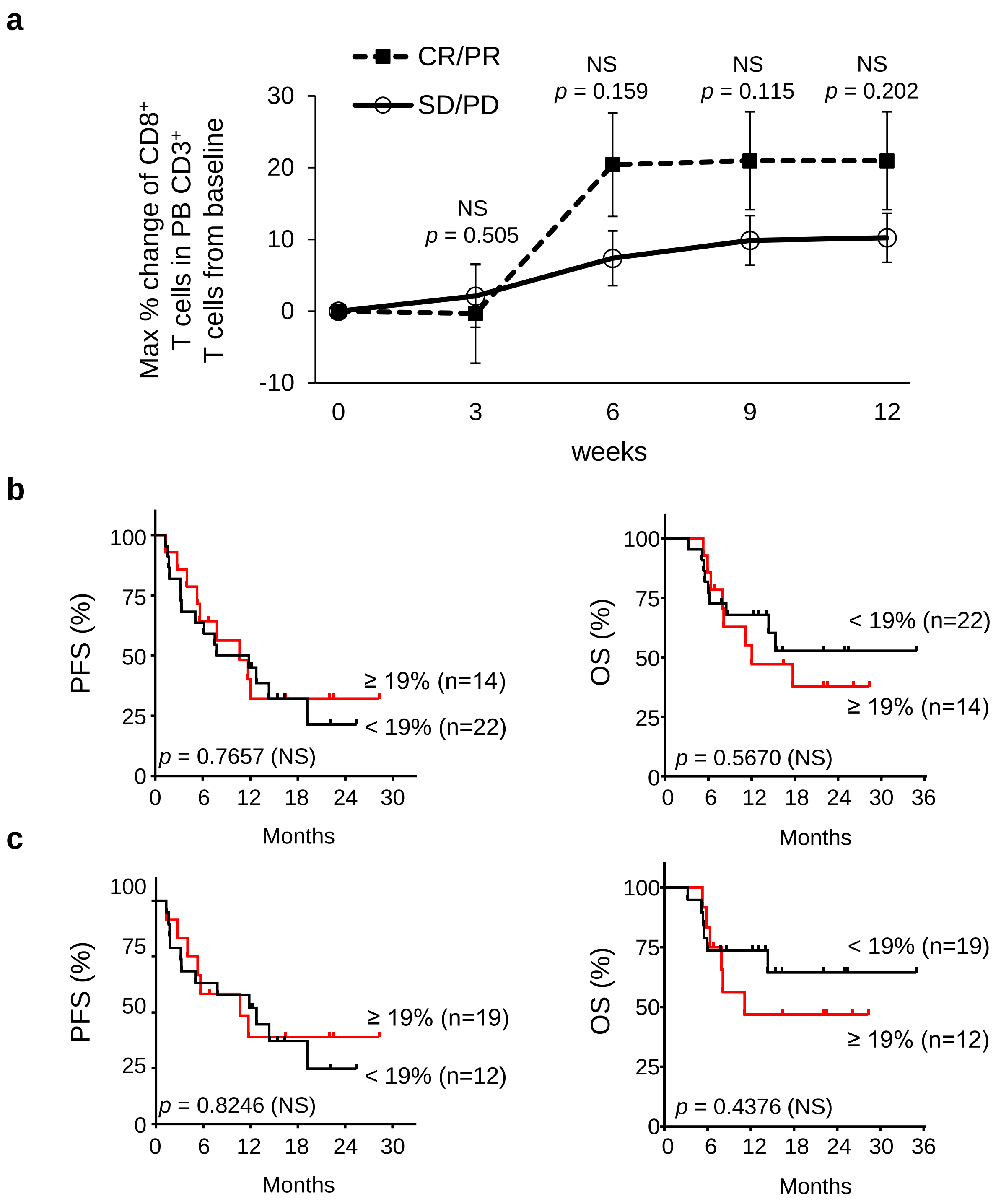

Supplemental Figure 10, Related to Figure 5.

a The largest % change of the $CD8^+$ T cells in PB $CD3^+$ T cells from baseline by the given time point in responders (CR/PR: n=13) and non-responders (SD/PD: n=23) of 36 NSCLC patients treated with anti-PD-1 therapy. CR/PR: complete and partial response, SD/PD: stable and progressive disease. NS, not significant by a two-tailed Mann-Whitney *U*-test. Values are median ± SEM.

b, c Kaplan-Meier progression free survival (PFS) and overall survival (OS) for high versus low % change of the $CD8^+$ T cells in PB $CD3^+$ T cells from baseline in all NSCLC patients (n=36) (b) and NSCLC patients with a PD-L1 TPS > 50% treated with Pembrolizumab (n=31) (c). *P* values were calculated by a log-rank (Mantel-Cox) test. NS, not significant. Source data are provided as a Source Data file.

Supplementary Table 1, Related to Figure 4 and Supplementary Figure 7

Six most dominant clones in sorted splenic CX3CR1$^+$ CD8$^+$ T cells (**a**) and CD8$^+$ TILs (**b**) in MC38 tumor-bearing mice treated with CTLA-4 and PD-L1 blockades (n=10 mice / experiment)

a

**Exp.**	**CDR3β region sequence**	**Frequency$^{\#}$ (%)**	**Sequence also found in CD8$^+$ TILs (%)**		
No. 1 (n=10)	CASSPRLGDNYAEQFF	4.26			
	CASSDRGRAEQFF	2.65	#1 (0.24)	#2 (2.19)	#3 (0.82)
	**CASSLVGNQDTQYF**	2.48	#1 (2.68)	#2 (9.68)	#3 (3.52)
	CASGDAQYNNQAPLF	2.23	#1 (1.82)		#3 (3.01)
	CASSPDKYEQYF	1.84			#3 (0.01)
	CASSQSGFAETLYF	1.75			
No. 2 (n=10)	CASSQWGAGNTLYF	17.59		#2 (1.44)	
	CASSPGRGYEQYF	2.92			
	CTCSPGTASGNTLYF	2.89		#2 (0.15)	
	**CASSLVGNQDTQYF**	2.38	#1 (2.68)	#2 (9.68)	#3 (3.52)
	CASSGRDRKNERLFF	1.87			
	CASGDSNERLFF	1.38	#1 (0.02)	#2 (0.75)	
No. 3 (n=10)	CAWRGTGSAETLYF	6.33			#3 (0.29)
	CASSGGRQYF	3.05			#3 (4.17)
	**CASSLVGNQDTQYF**	2.27	#1 (2.68)	#2 (9.68)	#3 (3.52)
	CASSNRVEQYF	1.87			#3 (0.005)
	CASRGDSYNYAEQFF	1.73			#3 (0.61)
	CASSPGRRSGNTLYF	1.47			#3 (0.22)

b

**Exp.**	**CDR3β region sequence**	**Frequency$^{\#}$ (%)**	**Sequence also found in CD8$^+$ TILs (%)**		
No. 1 (n=10)	CASTPRDWGVAEQFF	13.61			
	CASSRDLGNTGQLYF	7.25		#2 (2.63)	#3 (0.01)
	CASSLELGGPEQYF	3.84			#3 (0.01)
	CASSPGYAEQFF	3.68			
	CASSPGQGYAEQFF	3.52			#3 (1.01)
	**CASSLVGNQDTQYF**	2.68		#2 (9.68)	#3 (3.52)
No. 2 (n=10)	**CASSLVGNQDTQYF**	9.68	#1 (2.68)		#3 (3.52)
	CASRRTTNSDYTF	4.12			#3 (1.86)
	CASSSGTYEQYF	3.56			
	CASSLELGGREQYF	2.77	#1 (0.15)		
	CASSRDLGNTGQLYF	2.63	#1 (7.25)		#3 (0.01)
	CASHLSTSAETLYF	2.44			
No. 3 (n=10)	CTCSETGNSYEQYF	9.98			
	CASSGGRQYF	4.17			
	**CASSLVGNQDTQYF**	3.52	#1 (2.68)	#2 (9.68)	
	CASSGGWQYF	3.08			
	CASGDAQYNNQAPLF	3.01	#1 (1.81)		
	CASSPGQNYAEQFF	1.98	#1 (0.07)	#2 (0.20)	

$^{\#}$Productive frequency.

Bold font with yellow highlight indicates that the clone was present in splenic CX3CR1$^+$ CD8$^+$ T cells and CD8$^+$ TILs at high frequency (>2%) from all three independent experiments.

Green and blue-highlighted clones in sorted splenic CX3CR1$^+$ CD8$^+$ T cells (**a**) and CD8$^+$ TILs (**b**) have a high degree of sequence homology, respectively.

Supplementary Table 2, Related to Figure 5.
Demographic and clinical characteristics of lung cancer patients on this study

Patient characteristics	*n* = 36
Median age (range)	68 (49-89)
Sex, *n* (%)	
Male	14 (39%)
Female	22 (61%)
Race, *n* (%)	
Caucasian	34 (94%)
African-American	2 (6%)
ECOG PS	
0/1	33 (92%)
History of smoking	
Never	4 (11%)
Former	22 (61%)
Current	10 (28%)
Histology, *n* (%)	
Adenocarcinoma	22 (61%)
Squamous cell carcinoma	13 (36%)
Non-small cell lung cancer with giant features	1 (3%)
Stage at diagnosis, *n* (%)	
II-III	9 (25%)
IV	27 (75%)
Prior lung surgery, *n* (%)	6 (17%)
Prior chemotherapy, *n* (%)	
One line	6 (17%)
Two lines	3 (8%)
Prior targeted therapy, *n* (%)	
Osimertinib and Erlotinib (EGFR)	2 (6%)
Dabrafenib/Trametinib (BRAF V600E)	1 (3%)
Prior radiation, *n* (%)	
Thoracic radiation	16 (44%)
Bone radiation	4 (11%)
Gamma knife stereotactic radiosurgery	7 (19%)
Known brain metastases, *n* (%)	7 (19%)
Study drug, *n* (%)	
Nivolumab	2 (6%)
Pembrolizumab	34 (94%)
Best disease response at 12 weeks, *n* (%)	
Complete Response (CR)	0 (0%)
Partial response (PR)	13 (36%)
Stable disease (SD)	14 (3-96%)
Progressive disease (PD)	9 (25%)

PD-L1 expression tumor proportion score	
≥ 50%	31 (86%)
1-49%	5 (14%)
Tumor-infiltrating lymphocytes	
moderate/high infiltration	15 (42%)
no/minimal infiltration	9 (25%)
Tumor mutational burden	
High (≥ 10.0 mutations per megabase pairs)	5 (14%)
Low (< 10.0 mutations per megabase pairs)	17 (47%)

Supplementary Table 3, Related to Figure 5c.
Marker Performance

PB CX3CR1$^+$ CD8$^+$ T cells	Cut-point	Specificity	Sensitivity	PPV	NPV	AUC (95% CI)
Max % change by 3 weeks (n=27)	19.91	0.94	0.50	0.83	0.76	0.76 (0.55, 0.95)
Max % change by 6 weeks (n=36)	21.19	0.87	0.62	0.73	0.80	0.80 (0.53, 0.90)
Max % change by 9 weeks (n=36)	15.49	0.74	1.00	0.68	1.00	0.87 (0.74, 0.99)
Max % change by 12 weeks (n=36)	19.62	0.87	0.92	0.80	0.95	0.88 (0.78, 0.99)

Maximal percent change of the CX3CR1$^+$ subset in PB CD8$^+$ T cells at 3, 6, 9 and 12-weeks relative to baseline was evaluated. Cut-points for discriminating between responders and non-responders were obtained using the Youden's index criterion.
Abbreviations: PB, peripheral blood; Max, maximal; PPV, positive predictive value; NPV, negative predictive value.

Supplementary Table 4, Related to Figure 5 and Table 1.
Prediction performance for study biomarkers

	Baseline $CD8^+$ tumor-infiltrating lymphocytes (moderate/high vs. no/minimal infiltration) (n=24)	Baseline tumor mutational burden (≥10.0/Mbp vs. <10.0/Mbp) (n=22)
PPV	33.3% (5/15)	20.0% (1/5)
NPV	55.6% (5/9)	52.9% (9/17)
Sensitivity	55.6% (5/9)	11.1% (1/9)
Specificity	33.3% (5/15)	69.2% (9/13)
Accuracy	41.7% (10/24)	45.5% (10/22)

Abbreviations: PPV, positive predictive value; NPV, negative predictive value; Mbp, megabase pairs

Supplementary Table 5, Related to Figure 5 and Supplementary Figure 10.
Marker Performance

PB CD8$^+$ T cells	Cut-point	Specificity	Sensitivity	PPV	NPV	AUC (95% CI)
Max % change by 3 weeks (n=27)	1.76	0.59	0.70	0.5	0.77	0.58 (0.35, 0.81)
Max % change by 6 weeks (n=36)	19.08	0.78	0.54	0.58	0.75	0.65 (0.44, 0.85)
Max % change by 9 weeks (n=36)	19.08	0.78	0.62	0.62	0.78	0.66 (0.46, 0.86)
Max % change by 12 weeks (n=36)	19.08	0.74	0.62	0.57	0.77	0.60 (0.43, 0.83)

Maximal percent change of the CD8$^+$ T cells in PB CD3$^+$ T cells at 3, 6, 9 and 12-weeks relative to baseline was evaluated. Cut-points for discriminating between responders and non-responders were obtained using the Youden's index criterion.
Abbreviations: PB, peripheral blood; Max, maximal; PPV, positive predictive value; NPV, negative predictive value.

Supplementary Table 6: Antibody used for flow cytometry

Antibodies	Dilution	Source	Catlog Number
Anti-mouse CD8 alpha clone 53-6.7 BUV395	1:400	BD	Cat# 563786
Anti-mouse CD8 clone KT15 FITC	1:200	Thermo Fisher scientific	Cat# MA5-16759
Anti-mouse CD8 alpha clone 53-6.7 BV510	1:200	Biolegend	Cat# 100752
Anti-mouse CD62L clone MEL-14 BV421	1:200	Biolegend	Cat# 104436
Anti-mouse CXCR3 clone CXCR3-173 PerCP-Cy5.5	1:200	Biolegend	Cat# 126514
Anti-mouse CD3 clone 145-2C11 PerCP-Cy5.5	1:200	Biolegend	Cat# 100328
Anti-mouse CD45 clone 30-F11 Pacific Orange	1:200	Thermo Fisher scientific	Cat# MCD4530
Anti-mouse CD27 clone LG.7F9 PE-Cy7	1:200	Thermo Fisher scientific	25-0271-82
Anti-mouse CX3CR1 clone SA011F11 APC	1:200	Biolegend	Cat# 149008
Anti-mouse PD-1 clone 29F.1A12 BV711	1:200	Biolegend	Cat# 135231
Anti-mouse TIM3 clone RMT3-23 BV421	1:200	Biolegend	Cat# 119723
Anti-mouse CD4 clone GK1.5 FITC	1:200	Biolegend	Cat# 100406
Anti-mouse CD4 clone GK1.5 BUV737	1:200	BD	Cat# 564298
Anti-mouse 4-1BB clone 17B5 Biotin	1:200	Biolegend	Cat# 106104
Anti-mouse Thy1.2 clone 53-2.1 FITC	1:400	BD	Cat# 553003
Anti-mouse Thy1.2 clone 53-2.1 PerCP-Cy5.5	1:400	Biolegend	Cat# 140322
Anti-mouse Ki-67 clone 16A8 BV421	1:200	Biolegend	Cat# 652411
Anti-mouse KLRG1 clone 2F1 APC-Cy7	1:200	Biolegend	Cat# 138425
Anti-mouse GZMA clone GzA-3G8.5 PE	1:200	Thermo Fisher scientific	Cat# 12-5831-82
Streptavidin APC-Cy7	1:200	Biolegend	Cat# 405208
Streptavidin BV650	1:200	BD	Cat# 563855
H-2Db tetramer to peptide ASMTNMELM PE	1:100	NIH	N/A
H-2Ld tetramer to peptide SPSYVYHQF PE	1:100	NIH	N/A
Anti-mouse CD16/CD32 (Mouse BD Fc Block)	1:200	BD	Cat# 553141
Live/Dead Flexible Aqua Dead Cell Stain	1:200	Thermo Fisher scientific	L34966
Live/Dead Flexible Near-IR Dead Cell Stain	1:200	Thermo Fisher scientific	L34975
γ-Globulins from human blood	12 mg/ml	Sigma	G4386
Anti-human CX3CR1 clone 2A9-1 APC	1:100	Biolegend	Cat# 341610
Anti-human CD3 clone UCHT1 BB515	1:100	BD	Cat# 564465
Anti-human CD4 clone RPA-T4 PE	1:100	BD	Cat# 555347
Anti-human CD8 clone RPA-T8 BV 421	1:100	Biolegend	Cat# 301036
Anti-human CD8 clone 53-6.7 APC-eFluor 780	1:100	Thermo Fisher scientific	Cat# 47-0081-82
