# Supplementary material for: T-cell CX3CR1 expression as a dynamic blood-based biomarker of response to immune checkpoint inhibitors
Source: Nat Commun. 2021 Mar 3;12:1402. doi: 10.1038/s41467-021-21619-0 (PMC7930182; doi:10.1038/s41467-021-21619-0)
Supplement: Supplementary file 2 — Description of Additional Supplementary Files [file 41467_2021_21619_MOESM2_ESM.pdf]

**Description of Additional Supplementary Files**

**File Name: Supplementary Data 1.** CDR3β region amino acid sequence in sorted splenic CD27$^{lo}$ CX3CR1$^{-}$, CD27$^{hi}$ CX3CR1$^{-}$, and CX3CR1$^{+}$ CD8$^{+}$ T cells, and CD8$^{+}$ TILs in MC38 tumor-bearing mice treated with CTLA-4 and PD-L1 blockades (n=10 mice / experiment).
