# Supplementary material for: T-cell CX3CR1 expression as a dynamic blood-based biomarker of response to immune checkpoint inhibitors
Source: Nat Commun. 2021 Mar 3;12:1402. doi: 10.1038/s41467-021-21619-0 (PMC7930182; doi:10.1038/s41467-021-21619-0)
Supplement: Supplementary file 4 — Reporting Summary [file 41467_2021_21619_MOESM4_ESM.pdf]

Corresponding author(s): Fumito Ito

Last updated by author(s): Jan 27, 2021

Reporting Summary

Nature Research wishes to improve the reproducibility of the work that we publish. This form provides structure for consistency and transparency in reporting. For further information on Nature Research policies, see our Editorial Policies and the Editorial Policy Checklist.

Statistics

For all statistical analyses, confirm that the following items are present in the figure legend, table legend, main text, or Methods section.

n/a	Confirmed	
☐	☒	The exact sample size (n) for each experimental group/condition, given as a discrete number and unit of measurement
☐	☒	A statement on whether measurements were taken from distinct samples or whether the same sample was measured repeatedly
☐	☒	The statistical test(s) used AND whether they are one- or two-sided<br>*Only common tests should be described solely by name; describe more complex techniques in the Methods section.*
☒	☐	A description of all covariates tested
☐	☒	A description of any assumptions or corrections, such as tests of normality and adjustment for multiple comparisons
☐	☒	A full description of the statistical parameters including central tendency (e.g. means) or other basic estimates (e.g. regression coefficient) AND variation (e.g. standard deviation) or associated estimates of uncertainty (e.g. confidence intervals)
☐	☒	For null hypothesis testing, the test statistic (e.g. F, t, r) with confidence intervals, effect sizes, degrees of freedom and P value noted<br>*Give P values as exact values whenever suitable.*
☒	☐	For Bayesian analysis, information on the choice of priors and Markov chain Monte Carlo settings
☒	☐	For hierarchical and complex designs, identification of the appropriate level for tests and full reporting of outcomes
☒	☐	Estimates of effect sizes (e.g. Cohen's d, Pearson's r), indicating how they were calculated

Our web collection on statistics for biologists contains articles on many of the points above.

Software and code

Policy information about availability of computer code

Data collection	Flow cytometry data were acquired using BD FACSDiva v8.0.<br>TCRβ sequencing (TCRβ-Seq) was performed on an Illumina NextSeq system using 150 cycle mid-output kit (Illumina Inc.) with ImmunoSEQ immune profiling system at the survey level (Adaptive Biotechnologies).
Data analysis	All visualization and associated statistical analysis were performed using R v3.6.1 and GraphPad Prism v8.0.2.<br>Flow cytometric data analysis was performed using FlowJo v10.1.5.<br>TCRβ-Seq data analysis was performed using ImmunoSEQ Analyzer (v3.0), LymphoSeq (v1.16) and ImmunoMap v1.0.

For manuscripts utilizing custom algorithms or software that are central to the research but not yet described in published literature, software must be made available to editors and reviewers. We strongly encourage code deposition in a community repository (e.g. GitHub). See the Nature Research guidelines for submitting code & software for further information.

Data

Policy information about availability of data

All manuscripts must include a data availability statement. This statement should provide the following information, where applicable:
- Accession codes, unique identifiers, or web links for publicly available datasets
- A list of figures that have associated raw data
- A description of any restrictions on data availability

The TCR-seq data were deposited in the National Center for Biotechnology Information Gene Expression Omnibus (NCBI-GEO) under accession number GSE165383 at https://www.ncbi.nlm.nih.gov/geo/query/acc.cgi?acc=GSE165383, and on Zenodo at http://doi.org/10.5281/zenodo.4431045. The patients did not give their consent for the public availability of their raw sequencing data. DNA sequencing data from lung cancer patients are available with a data share agreement, and can be requested from F.I. (fumito.ito@roswellpark.org). Any other relevant data are available from the corresponding author upon reasonable request. The source

data underlying graphs in Figure 1–5 and Supplementary Figure 2, 3, 9 and 10 has been provided as a source data file.

Field-specific reporting

Please select the one below that is the best fit for your research. If you are not sure, read the appropriate sections before making your selection.

- [x] Life sciences
- [ ] Behavioural & social sciences
- [ ] Ecological, evolutionary & environmental sciences

For a reference copy of the document with all sections, see nature.com/documents/nr-reporting-summary-flat.pdf

Life sciences study design

All studies must disclose on these points even when the disclosure is negative.

Sample size	[Pre-clinical study] Sample sizes were determined based on our and other investigators experience with the respective cell lines used (e.g.: Saito et al. 2016 (PMID: 27197199), Yamauchi et al. 2020 (PMID: 32255766), Oba et al. 2020 (PMID: 32848036), Ngiow et al 2015 (PMID: 26208901), Juneja et al. 2017 (PMID: 28302645)). No statistical methods were used as we observed many statistically significant effects in the data with the above methods of sample size selection without a priori sample size calculations.<br>[Clinical study] No statistical methods were used to predetermine sample size. The clinical samples are selected based on availability during the study window. The observed sample size provides a sufficient pool of both responders and non-responders, and produces performance measures (i.e. AUC, sensitivity, etc.) with adequate levels of precision.
Data exclusions	No data were excluded from the analyses.
Replication	All findings reported were reproducible and data shown are pooled from >=2 independent experiments, with comparable results in each experiment.
Randomization	Randomization only relevant for experiments involving animal models. 6- to 12-week old mice were matched by age and sex and randomly assigned to specific treatment groups. For all experiments with tumor-bearing mice, groups were randomized based on tumor size prior to treatment start so that all cages had mice with tumors of similar average size. For clinical study, randomization was not appropriate or feasible due to the nature of this study. We examined the association between a biomarker measured over time and response.
Blinding	[Pre-clinical study] TCRseq analyses were performed in a blinded fashion. Investigators were not blinded to group allocation during data collection and analysis for experimental mouse interventions as knowledge of the treatment groups was required.<br>[Clinical study] Clinical response was determined by medical oncologists who do not know the biomarker performance (flow cytometry data).

Reporting for specific materials, systems and methods

We require information from authors about some types of materials, experimental systems and methods used in many studies. Here, indicate whether each material, system or method listed is relevant to your study. If you are not sure if a list item applies to your research, read the appropriate section before selecting a response.

Materials & experimental systems

n/a	Involved in the study	
☐	☒	Antibodies
☐	☒	Eukaryotic cell lines
☒	☐	Palaeontology and archaeology
☐	☒	Animals and other organisms
☐	☒	Human research participants
☒	☐	Clinical data
☒	☐	Dual use research of concern

Methods

n/a	Involved in the study	
☒	☐	ChIP-seq
☐	☒	Flow cytometry
☒	☐	MRI-based neuroimaging

Antibodies

Antibodies used	Anti-mouse mouse antibodies:<br>All surface anti-mouse antibodies were used at 1:200, except for CD8a (clone 53-6.7 BUV395, #563786) and Thy1.2 (1:400); all intracellular antibodies were used at 1:200.<br>Biolegend: CD3 (clone 145-2C11 PerCP/Cyanine5.5, #100328), CD90.2 (clone 53-2.1 PerCP-Cy5.5, #140322), CD4 (clone GK1.5 FITC, # 100406), CD8a (clone 53-6.7 BV510, #100752), CX3CR1 (clone SA011F11 APC, #149008), CD62L (clone MEL-14 BV421, #104436), CXCR3 (clone CXCR3-173 PerCP-Cy5.5, #126514), PD-1 (clone 29F.1A12 BV711, #135231), 4-1BB (clone 17B5 Biotin, #106104), Ki67 (clone 16A8 BV421, #652411), KLRG1(clone 2F1 APC-Cy7, #138425), TIM3 (clone RMT3-23, BV421, #119723), Streptavidin APC-Cy7 (#405208).<br>BD: CD8a (clone 53-6.7 BUV395, #563786), CD4 (clone GK1.5 BUV737, # 564298), CD90.2 (clone 53-2.1 FITC, #553003), Streptavidin BV650 (#563855), CD16/CD32 (#553141).<br>Thermo Fisher scientific: CD27 (clone LG.7F9 PE-Cy7, #25-0271-82), CD45 (clone 30-F11 Pacific orange, #MCD4530), CD8 (clone KT15

FITC, #MA5-16759), GZMA (clone GzA-3G8.5 PE, #12-5831-82), Live/Dead Flexible Aqua Dead Cell Stain (#L34966), Live/Dead Flexible Near-IR Dead Cell Stain (#L34975).

Anti-human antibodies:
Biolegend: CX3CR1 (clone 2A9-1 APC, #341610) dilution 1:100, CD8 (clone RPA-T8 BV 421, #301036) dilution 1:100
BD: CD3 (clone UCHT1 BB515, #564465) dilution 1:100, CD4 (clone RPA-T4 PE, #555347) dilution 1:100
Thermo Fisher scientific: CD8 (clone 53-6.7 APC-eFluor 780, #47-0081-82) dilution 1:100
Sigma: γ-Globulins from human blood (#G4386).
Agilent: anti-CD8 antibody for IHC (clone C8/144B) dilution 1:200

In vivo treatment:
BioXCell: PD-L1 (clone 10F.9G2, # BE0101), CTLA-4 (clone 9H10, # BE0131).

Validation

Antibodies employed in our study were validated by the manufacturers and used according to the manufacturer's instructions or as used in recent publications from our group as evidenced in our recent publications, Yamauchi T.,et al., JCI insight 2020 (PMID: 32255766), Oba et al. JI 2020 (PMID: 32848036), and Oba et al. Nature Communications 2020 (PMID: 33110069)

Anti-mouse antibodies:
Biolegend: CD3 (clone 145-2C11 PerCP/Cyanine5.5, #100328, https://www.biolegend.com/en-us/products/percp-cyanine5-5-anti-mouse-cd3epsilon-antibody-4191), CD90.2 (clone 53-2.1 PerCP-Cy5.5, #140322, https://www.biolegend.com/en-us/products/percp-cyanine5-5-anti-mouse-cd902-thy-12-antibody-8993), CD4 (clone GK1.5 FITC, # 100406, https://www.biolegend.com/en-us/products/fitc-anti-mouse-cd4-antibody-248), CD8a (clone 53-6.7 BV510, #100752, https://www.biolegend.com/en-us/products/brilliant-violet-510-anti-mouse-cd8a-antibody-7992), CX3CR1 (clone SA011F11 APC, #149008, https://www.biolegend.com/en-us/products/apc-anti-mouse-cx3cr1-antibody-10460), CD62L (clone MEL-14 BV421, #104436, https://www.biolegend.com/en-us/products/brilliant-violet-421-anti-mouse-cd62l-antibody-7164), CXCR3 (clone CXCR3-173 PerCP-Cy5.5, #126514, https://www.biolegend.com/en-us/products/percp-cyanine5-5-anti-mouse-cd183-cxcr3-antibody-5020), PD-1 (clone 29F.1A12 BV711, #135231, https://www.biolegend.com/en-us/products/brilliant-violet-711-anti-mouse-cd279-pd-1-antibody-12303), 4-1BB (clone 17B5 Biotin, #106104, https://www.biolegend.com/en-us/products/biotin-anti-mouse-cd137-antibody-49), Ki67 (clone 16A8 BV421, #652411, https://www.biolegend.com/en-us/products/brilliant-violet-421-anti-mouse-ki-67-antibody-8982), KLRG1(clone 2F1 APC-Cy7, #138425, https://www.biolegend.com/en-us/products/apc-cyanine7-anti-mouse-human-klrg1-mafa-antibody-12486), TIM3 (clone RMT3-23, BV421, #119723, https://www.biolegend.com/en-us/products/brilliant-violet-421-anti-mouse-cd366-tim-3-antibody-13392), Streptavidin APC-Cy7 (#405208, https://www.biolegend.com/en-us/products/apc-cyanine7-streptavidin-1471).
BD: CD8a (clone 53-6.7 BUV395, #563786, https://www.bdbiosciences.com/us/reagents/research/antibodies-buffers/immunology-reagents/anti-mouse-antibodies/cell-surface-antigens/buv395-rat-anti-mouse-cd8a-53-67/p/563786), CD4 (clone GK1.5 BUV737, # 564298), https://www.bdbiosciences.com/us/reagents/research/antibodies-buffers/immunology-reagents/anti-mouse-antibodies/cell-surface-antigens/buv737-rat-anti-mouse-cd4-gk15/p/612761 CD90.2 (clone 53-2.1 FITC, #553003, https://www.bdbiosciences.com/us/applications/research/stem-cell-research/cancer-research/mouse/fitc-rat-anti-mouse-cd902-53-21/p/553003), Streptavidin BV650 (#563855, https://www.bdbiosciences.com/us/reagents/research/antibodies-buffers/second-step-reagents/avidinstreptavidin/bv650-streptavidin/p/563855), CD16/CD32 (#553141, https://www.bdbiosciences.com/us/applications/research/b-cell-research/surface-markers/mouse/purified-rat-anti-mouse-cd16cd32-mouse-bd-fc-block-24g2/p/553141).
Thermo Fisher scientific: CD27 (clone LG.7F9 PE-Cy7, #25-0271-82, https://www.thermofisher.com/antibody/product/CD27-Antibody-clone-LG-7F9-Monoclonal/25-0271-82), CD45 (clone 30-F11 Pacific orange, #MCD4530, https://www.thermofisher.com/antibody/product/CD45-Antibody-clone-30-F11-Monoclonal/MCD4530), CD8 (clone KT15 FITC, #MA5-16759, https://www.thermofisher.com/antibody/product/CD8-alpha-Antibody-clone-KT15-Monoclonal/MA5-16759), GZMA (clone GzA-3G8.5 PE, #12-5831-82, https://www.thermofisher.com/antibody/product/Granzyme-A-Antibody-clone-GzA-3G8-5-Monoclonal/12-5831-82), Live/Dead Flexible Aqua Dead Cell Stain (#L34966, https://www.thermofisher.com/order/catalog/product/L34966#/L34966), Live/Dead Flexible Near-IR Dead Cell Stain (#L34975, https://www.thermofisher.com/order/catalog/product/L34975?SID=srch-hj-L34975#/L34975?SID=srch-hj-L34975).

Anti-human antibodies:
Biolegend: CX3CR1 (clone 2A9-1 APC, #341610, https://www.biolegend.com/en-us/products/apc-anti-human-cx3cr1-antibody-6605), CD8 (clone RPA-T8 BV 421, #301036, https://www.biolegend.com/en-us/products/brilliant-violet-421-anti-human-cd8a-antibody-7152).
BD: CD3 (clone UCHT1 BB515, #564465, https://www.bdbiosciences.com/us/applications/research/t-cell-immunology/th-1-cells/surface-markers/human/bb515-mouse-anti-human-cd3-ucht1-also-known-as-ucht-1-ucht-1/p/564465), CD4 (clone RPA-T4 PE, #555347, https://www.bdbiosciences.com/us/applications/research/t-cell-immunology/th-1-cells/surface-markers/human/pe-mouse-anti-human-cd4-rpa-t4/p/555347).
Thermo Fisher scientific: CD8 (clone 53-6.7 APC-eFluor 780, #47-0081-82, https://www.thermofisher.com/antibody/product/CD8a-Antibody-clone-53-6-7-Monoclonal/47-0081-82).
Sigma: γ-Globulins from human blood (#G4386, https://www.sigmaaldrich.com/catalog/product/sigma/g4386?lang=en&region=US).
Agilent: anti-CD8 antibody for IHC (clone C8/144B), https://www.agilent.com/en/product/immunohistochemistry/antibodies-controls/primary-antibodies/cd8-(concentrate)-76631

In vivo treatment:
BioXCell: PD-L1 (clone 10F.9G2, # BE0101, https://bxcell.com/product/m-pdl-1/), CTLA-4 (clone 9H10, # BE0131, https://bxcell.com/product/m-cd152-m-ctla-4/).

Eukaryotic cell lines

Policy information about cell lines

Cell line source(s)

MC38 and CT26 murine colon adenocarcinoma cell lines were gifts from Dr. Weiping Zou (University of Michigan) and Dr.

	Sharon Evans (Roswell Park Comprehensive Cancer Center), respectively. B16F10 (B16) melanoma cell line was purchased from ATCC.
Authentication	Cell lines obtained from external institutions were authenticated by morphology, phenotype and growth.
Mycoplasma contamination	Cells lines tested negative for mycoplasma contamination prior to sample generation. Samples were confirmed negative using MycoAlert (Lonza) Mycoplasma Detection Kit.
Commonly misidentified lines (See ICLAC register)	No commonly misidentified lines were used in this study.

Animals and other organisms

Policy information about studies involving animals; ARRIVE guidelines recommended for reporting animal research

Laboratory animals	Male and female C57BL/6 mice and female Balb/c mice were purchased from the Jackson Laboratories. All mice were 7 to 12 weeks old at the beginning of each experiment, and were housed in the Unit for Laboratory Animal Medicine at the Roswell Park Comprehensive Cancer Center in compliance with the Institutional Animal Care and Use Committee regulations. Housing conditions at the Roswell Park Comprehensive Cancer Center: Mice were maintained in a specific pathogen free unit on a 12hr light: 12hr dark cycle. The animal rooms are provided with 100% fresh, HEPA filtered air at 10-15 air changes per hour. Room temperatures are controlled by reheat units within each room, and are maintained within the range of 70°F ± 2° F. The Humidity levels are controlled globally, and it is maintained between 30-70%.
Wild animals	No wild animals were used in this study.
Field-collected samples	No field-collected samples were used in this study.
Ethics oversight	All experiments were reviewed and approved by the Institutional Animal Care and Use Committee of the institute at Roswell Park Comprehensive Cancer Center.

Note that full information on the approval of the study protocol must also be provided in the manuscript.

Human research participants

Policy information about studies involving human research participants

Population characteristics	Non-small cell lung cancer (NSCLC) patients were recruited irrespective of gender and were recruited on the basis of age (>18 y.o.) and treatment (anti-PD-1 therapy). Thirty-six patients with PD-L1 IHC positive NSCLC who underwent at least one full dose of anti-PD-1 Ab, blood collection at baseline and post-treatment, and imaging studies were evaluated. The cohort includes 14 male and 22 female patients, (34 Caucasian and 2 African-American) at median age of 68 (49-89).
Recruitment	NSCLC patients undergoing treatment with anti-PD-1 therapy were recruited from Thoracic Medicine Oncology clinic. There was no potential for self-selection bias or any other bias.
Ethics oversight	Human experimental work was conducted according to the Declaration of Helsinki Principles. All patients were consented to the collection and storage of blood samples, the analysis of archived tumor tissue, and the review of their medical records under the protocol (I 188310), in accordance with the Institutional Review Board of Roswell Park Comprehensive Cancer Center. Human Subject Assurance Number: 00006731.

Note that full information on the approval of the study protocol must also be provided in the manuscript.

Flow Cytometry

Plots

Confirm that:

- [x] The axis labels state the marker and fluorochrome used (e.g. CD4-FITC).
- [x] The axis scales are clearly visible. Include numbers along axes only for bottom left plot of group (a 'group' is an analysis of identical markers).
- [x] All plots are contour plots with outliers or pseudocolor plots.
- [x] A numerical value for number of cells or percentage (with statistics) is provided.

Methodology

Sample preparation	Sample preparation is described in details in the Methods section.
Instrument	Samples were acquired using LSR II (BD), LSRFortessa (BD), BD FACSARIA II or SONY SH800 sorter.
Software	FACSDIVA software v8.0 (BD Biosciences) was used for acquisition. FlowJo software v10.1.5 (TreeStar) was used for analysis
Cell population abundance	Dead cells and doublets were excluded on the basis of forward and side scatter and Fixable Live/Dead NearIR. Purity of sorted cells was >95% as shown in the Supplementary Figure 5. CD8+ tumor-infiltrating lymphocytes were immunomagnetically

sorted by Miltenyi method as described in details in the Methods section.

Gating strategy

Gating strategy is described in the results section of the manuscript (Figures 2, 3, 5 and Supplementary Figure 1 for (Figures 1, 3, and 4, and Supplementary Figures 2 – 5). Cells were gated on FSC-A v SSC-A for lymphocytes, FSC-H v FSC-A for singlets, Live/dead cell discrimination was performed using Live/Dead Fixable Aqua Dead Cell Stain Kit or LIVE/DEAD Fixable Near-IR Dead Cell Stain Kit (Invitrogen).

☒ Tick this box to confirm that a figure exemplifying the gating strategy is provided in the Supplementary Information.
